# Prognostic Landscape of Tumor-Infiltrating T and B Cells in Human Cancer

**DOI:** 10.3389/fimmu.2021.731329

**Published:** 2022-01-04

**Authors:** Ming Zheng, Yi-Ming Li, Zhen-Yu Liu, Xin Zhang, Yinghui Zhou, Jian-Li Jiang, Ping Zhu, Xiang-Min Yang, Juan Tang, Zhi-Nan Chen

**Affiliations:** ^1^Institute of Military Cognition and Brain Sciences, Academy of Military Medical Sciences, Beijing, China; ^2^Beijing Institute of Basic Medical Sciences, Beijing, China; ^3^State Key Laboratory of Cancer Biology, Cell Engineering Research Center and Department of Cell Biology, Fourth Military Medical University, Xi’an, China; ^4^National Translational Science Center for Molecular Medicine, Xi’an, China; ^5^Department of Clinical Immunology, Xijing Hospital, Fourth Military Medical University, Xi’an, China

**Keywords:** tumor-infiltrating lymphocytes, tumor-infiltrating B cells, tumor-infiltrating T cells, cancer, prognosis, single-cell RNA-sequencing, tumor microenvironment

## Abstract

Recently, immunotherapy targeting tumor-infiltrating lymphocytes (TILs) has emerged as a critical and promising treatment in several types of cancer. However, not all cancer types have been tested in immunotherapeutic trials, and different patients and cancer types may have unpredictable clinical outcomes. This situation has created a particular exigency for analyzing the prognostic significance of tumor-infiltrating T cells (TIL-T) and B cells (TIL-B) across different cancer types. To address the critical role of TILs, the abundances of TIL-T and TIL-B cells, as determined by the protein levels of LCK and CD20, were analyzed across heterogeneous human malignancies. TIL-T and TIL-B cells showed varying prognostic significances across heterogeneous cancer types. Additionally, distinct distributions of TIL-T and TIL-B cells were observed in different cancer and tumor microenvironment (TME) subtypes. Next, we analyzed the cellular context for the TME communication network involving the well-acknowledgeable chemokine receptors of TIL-T and TIL-B cells, implying the functional interactions with TME. Additionally, these chemokine receptors, expressed by TIL-T and TIL-B cells, were remarkably correlated with the levels of TIL-T or TIL-B cell infiltrations across nearly all the cancer types, indicating these chemokine receptors as universal targets for up- and down-regulating the TIL-T and TIL-B cells. Lastly, we provide the prognostic landscape of TIL-T and TIL-B cells across 30 cancer types and the subgroups defined by gender, histopathology, histological grade, therapeutic approach, drug, and TME subtype, which are intended to be a resource to fuel the investigations of TILs, with important implications for cancer immunotherapy.

## Introduction

Recent advances in the tumor microenvironment (TME) have demonstrated the complex interplay between the tumor and the adaptive immune cells (1). The adaptive immune response to tumors primarily relies on the tumor-infiltrating lymphocytes (TILs). TILs, mainly composed of T and B cells, prominently impact cancer patients’ survival and treatment outcomes (2–5). Recently, immunotherapy targeting TILs has emerged as a critical and promising treatment in several types of cancer (6–8). However, not all cancer types have been tested in immunotherapeutic trials, and different patients and cancer types may have unpredictable clinical outcomes. This situation has created a particular exigency for analyzing the prognostic significance of TILs across different cancer types.

Previous studies have reported the evaluation of immune cell abundances using bioinformatic approaches that analyze the expression of cell markers at the transcriptional level (9). However, it is reported that the transcriptome shows a low correlation to the proteome (10). Since transcriptional expression levels are imprecisely reflective of protein abundance, the analysis of TILs’ abundance need to be corroborated by approaches quantifying protein expression. Besides using bioinformatic approaches on transcriptional data, several clinical studies based on immunochemistry (IHC) have analyzed the abundances of tumor-infiltrating T cells (TIL-T) and B cells (TIL-B) through the protein levels of CD3 and CD20 (11–15). However, to assess the clinical relevance, these studies choose predefined cut-offs to divide the patients into subgroups. The predefined cut-off is unable to convey the prognostic relevance across the continuous gradient of TILs comprehensively. Besides, different studies usually use different cut-offs, such as median, tertile, and quartile cut-offs, which makes it difficult to analyze those fragmented studies systematically.

To fill these knowledge gaps, firstly, we conducted the protein-expression-based measurement for the abundances of TILs. Secondly, we employed a novel analysis framework to evaluate the prognostic significance of TILs towards the favorable and unfavorable outcomes by exhaustively considering all the continuous cut-offs. Thirdly, using the novel prognostic evaluation methodology, we analyzed the prognostic relevance of TIL-T and TIL-B cells in 7,694 cancer tissues across 32 human cancer types systematically.

A previous study has reported that the abundances of TILs are dramatically shaped by the TME, which could be classified into six distinct subtypes, including wound healing, IFN-γ dominant, inflammatory, lymphocyte depleted, immunologically quiet, and TGF-β dominant subtypes (1). In our study, we also investigated the distributions of TIL-T and TIL-B cells across different TME subtypes. Furthermore, through analyzing the expression of well-acknowledgeable receptor-ligand pairs using the single-cell RNA-sequencing (sc-RNAseq) data, we constructed the receptor-ligand network and inferred the communications between the TME and TILs.

Moreover, in order to provide a high-resolution landscape of TILs, we characterized the abundances and the prognostic associations of TIL-T and TIL-B cells across different clinical, therapeutic, histological, and TME subgroups. The detailed information of TILs’ prevalence and prognosis for these subgroups were provided in this study, which was intended to serve as a resource to fuel further studies of TILs, with important implications for the TILs-based immunotherapies.

## Materials and Methods

### Patient Samples

In this study, the TCGA mRNA, RPPA, clinical, immune subtype, and CIBERSORT data were obtained from the legacy archive of the GDC (https://portal.gdc.cancer.gov/legacy- archive/search/) and the TCGA publication page (https://gdc.cancer.gov/about-data/publications/pancanatlas). The TCGA data were standardized, normalized and batch corrected by the PanCancer Atlas consortium (16, 17). The TCGA mRNA data included 11,539 samples of 35 cancer types, and the TCGA RPPA data included 7,694 samples of 32 cancer types. For the TCGA RPPA data, tumor types with more than 30 samples were used for further analysis, and 30 cancer types met the criteria. ImmunePRECOG data was obtained from PRECOG (http://precog.stanford.edu) (18). All the data in this study were derived from previously published studies. Thus, the ethics approval and consent to participate were not applicable.

### GTEx and CCLE Data Availability and Analysis

In this study, the Genotype-Tissue Expression (GTEx) RNA-sequencing dataset of 8555 human tissue samples was obtained from GTEx Portal (https://gtexportal.org) (19). The Cancer Cell Line Encyclopedia (CCLE) RPPA data of 899 cell lines were obtained from CCLE (https://data.broadinstitute.org/ccle/) (20).

### Single-Cell RNA-Sequencing Data Availability and Analysis

The 52K lung cancer single-cell data was obtained in ArrayExpress under accessions E-MTAB-6149 and E-MTAB-6653 (21). The authors generously provided the detailed lineage annotations for each cell. The 10K human PBMC data were obtained from the 10x Genomics (https://support.10xgenomics.com/single-cell-gene-expression/datasets/3.0.0/pbmc_10k_protein_v3). The 33K human PBMC data was obtained from the Satija lab (https://satijalab.org/seurat/get_started_v1_4.html). The data were then log-transformed before further downstream analysis using Seurat (https://github.com/satijalab/seurat/). The single-cell data analysis code is available at https://github.com/10XGenomics/.

### Prognostic Association Analysis

The survival analysis was performed by the survival and survivALL package (22) in R v3.5.1. The protein expression levels were binned into two groups according to all the possible cutoffs (each group below or above the cutoff should have at least 15 patients). For each possible cutoff, the multivariate Cox proportional-hazards regression analysis was performed, with covariates including age, gender, histological grade, and stage (if applicable), to evaluate 5-year OS (overall survival), PFI (progression-free interval), DFI (disease-free survival), and DSS (disease-specific survival) in each cancer types and the subgroups defined by gender, histopathology, histological grade, therapeutic approach, drug, and TME subtype.

### Statistical Analysis

All statistical analyses were performed by using R v3.5.1. Non-parametric (Mann–Whitney test or Wilcoxon signed-rank for two samples and Friedman or Kruskal–Wallis with Dunn’s multiple comparison test for multiple samples), parametric (unpaired t-test for two samples or ordinary one-way ANOVA with Tukey’s multiple comparison test for multiple samples) tests, and Spearman correlation were performed using the stats package as appropriate. All statistical tests used 0.05 as the significance level, and *p* < 0.05 were considered as significant difference, indicated with an asterisk (*p < 0.05, **p < 0.01, ***p < 0.001 and ****p < 0.0001).

## Results

### CD20 and LCK as Specific Cell Markers for B and T Cells

**Figure 1:**
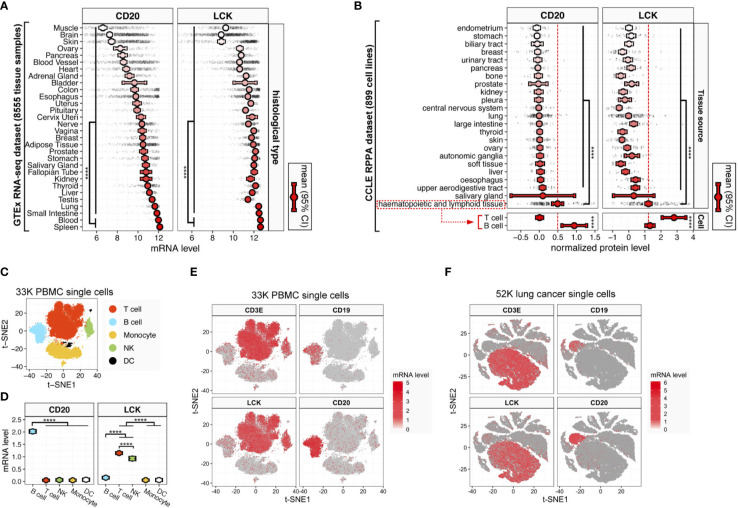
CD20 and LCK expression in different human tissue, cell, and cancer types. **(A)** CD20 and LCK mRNA expression in human tissues. **(B)** CD20 and LCK protein expression in human cell lines. Error bars showing mean ± 95% confidence interval (CI). The *p*-value was calculated using ANOVA. (*p-value<0.05, **p -value<0.01, ***p-value<0.001, ****p-value<0.0001) **(C)** t-SNE projection of single-cell RNA-sequencing data from 28,823 human PBMCs (known as 33K PBMCs dataset), with each dot representing one single cell and colors representing the five major cell lineages. The cell lineages were assigned according to the expression of known canonical marker genes (also see **Figure S1**). **(D)** LCK and CD20 mRNA expression in five major cell lineages. Error bars show mean ± 95% CI. Statistics were computed using ANOVA. **(E, F)** The t-SNE projection of 28,823 PBMCs **(E)** and 52,698 lung cancer [**(F)**, known as 52K lung cancer dataset; also see **Figure S5**], color-coded according to the expression of LCK, CD3E, CD19, and CD20 in each subgraph.

To construct the protein-level measurement of TIL infiltration, we looked through the TCGA reverse-phase protein arrays (RPPA) data (23). In the TCGA RPPA data of approximately 200 proteins across 32 cancer types, the most probable cell markers for B and T cells were CD20 and LCK. Here, we analyzed the specificity of CD20 and LCK as cell markers for B and T cells. In the Genotype-Tissue Expression (GTEx) RNA-sequencing dataset, the CD20 and LCK mRNA levels were significantly higher in the blood and spleen tissues, which are the hematopoietic and lymphoid tissues (**Figure 1A**). Next, in the Cancer Cell Line Encyclopedia (CCLE) RPPA dataset, we found that the CD20 and LCK proteins expressed dominantly in cell lines derived from the hematopoietic and lymphoid tissues (**Figure 1B**, upper plots). The CD20 and LCK protein levels were prominently higher in the cell lines derived from B and T cells, respectively (**Figure 1B**, lower plots).

Next, using the sc-RNAseq data of 28,823 human PBMCs (referred to as 33K PBMC dataset) with K-means clustering visualized in the two-dimensional projection of t-distributed stochastic neighbour embedding (t-SNE), we identified five distinct cell subtypes using canonical markers of major cell types, including T cells, B cells, monocytes, nature killer (NK) cells, and dendritic cells (DCs) (**Figures 1C** and **S1**). CD20 was dominantly expressed in B cells, while LCK was dominantly expressed in both NK cells and T cells (**Figures 1D, E**). Next, to clarify the relative distributions of LCK-expressing cells that include T and NK cells, we analyzed the CIBERSORT ImmunePRECOG dataset included 7,741 samples from 25 cancer types (**Figure S2**) and the CIBERSORT TCGA dataset included 11,273 samples from 33 cancer types (**Figure S3**). We found that the abundances of T cells were averagely 8 to 100-fold higher than that of NK cells (**Figures S2–3**). The LCK-expressing cells were significantly dominated by the T cells.

To further validate the above findings, we added two independent sc-RNAseq datasets, including the 10K PBMC dataset of 7,865 human PBMCs (**Figure S4**) and 52K lung cancer dataset of 52,698 cells from human lung cancer tissues (**Figures 1F** and **S5**). We found that, at the single-cell level, the expression of LCK and CD20 were largely consistent with the well-acknowledged T and B cell markers, CD3 and CD19 (**Figures 1E, F** and **Figure S4**). Moreover, in the TCGA RNA-seq dataset of 35 cancer types, we observed significant correlations between the transcript levels of CD3E and LCK (**Figure S6A**, pan-cancer rho=0.89), and the CD19 and CD20 (**Figure S6B**, pan-cancer rho=0.70) across different cancer types, indicating LCK and CD20 as specific cell markers for the T and B cells.

### Varying Distributions of Tumor-Infiltrating T and B Cells (TIL-T and TIL-B) Across Different Cancer Types

**Figure 2:**
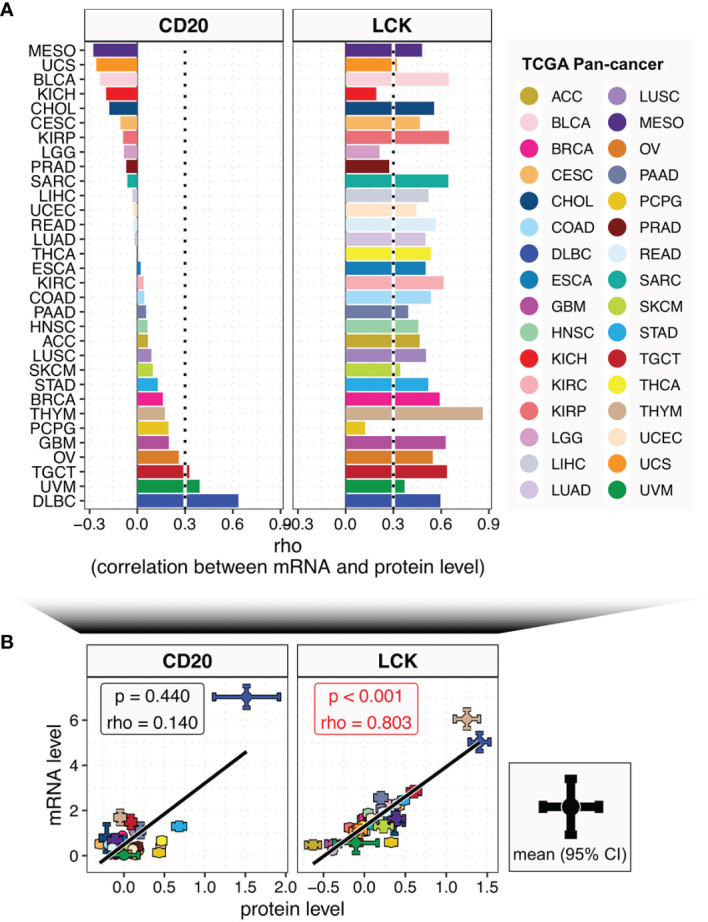
The relationships between the mRNA and protein levels of CD20 and LCK across different cancer types. **(A)** Bar plot shows the Spearman’s rho coefficients between mRNA and protein levels of CD20 and LCK in different cancer types, with color coding according to cancer type. **(B)** The correlations between mRNA and protein levels of CD20 and LCK in pan-cancer analysis. The error bar represents the 95% confidence interval (CI), the dot represents the average value, with color coding according to cancer type.

The evaluation of TILs’ abundances based on the TCGA RNA-seq data has been reported previously (9, 18). However, it is reported that the transcriptome only has a low correlation to the proteome (10). To investigate this phenomenon across different cancer and tissue types, we compared the correlations between the mRNA and protein levels of CD20 and LCK in the TCGA (**Figure 2**) and CCLE (**Figure S7**) datasets. We found that: (i) the correlation levels for both CD20 and LCK were varying across different cancer and tissue types (**Figures 2A** and **S7A**); (ii) in both the pan-cancer and pan-tissue analyses, the associations were significant in LCK but insignificant in CD20 (**Figures 2B** and **S7B**); (iii) the correlation levels for CD20 were very low in 93.8% of the cancer types and 91.3% of the tissue types (**Figures 2A** and **S7A**; Spearman rho < 0.3); by contrast, the low correlation levels for LCK were observed in 12.5% of cancer types and 60.9% of tissue types. Moreover, except for THYM, none of the cancer types showed a high correlation coefficient of Spearman rho > 0.7. These findings revealed the discordance of the mRNA-protein relationship, indicating the importance of protein-expression-based measurement of TILs’ abundances.

**Figure 3:**
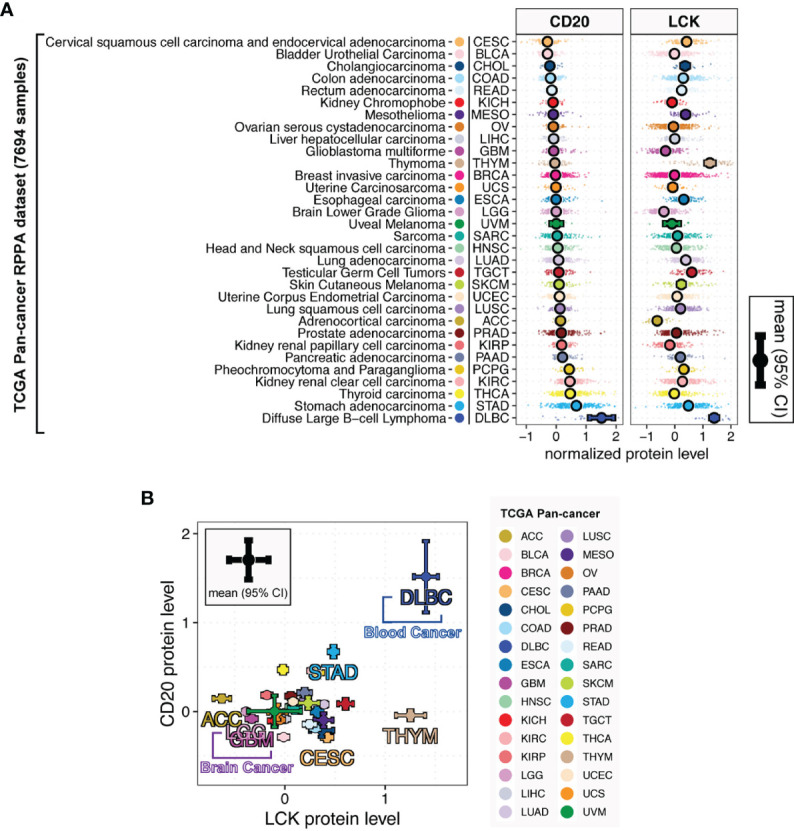
LCK and CD20 protein levels across different cancer types. **(A)** The protein levels of CD20 and LCK across different cancer types. **(B)** The relationship between the CD20 and LCK protein levels. The error bar represents the 95% confidence interval (CI), the dot represents the average value, with color coding according to cancer type.

Next, we analyzed the TIL-B and TIL-T abundances using the CD20 and LCK protein levels in the TCGA RPPA dataset, including 7,694 primary cancer tissues across 32 cancer types (**Figure S8**). The TCGA RPPA data has been previously harmonized by the PanCanAtlas consortium for uniform quality control, batch effect correction, and normalization (16). To the best of our knowledge, it is the first large-scale pan-cancer study of TIL-B and TIL-T cells using the protein-expression-based measurement. Here, **Figure 3A** shows the substantially varying distributions of LCK and CD20 protein levels across different cancer types. In the direct comparisons across 32 cancer types, the expression levels of CD20 and LCK in DLBC (lymphoid neoplasm diffuse large B cell lymphoma) were first and second-highest, respectively, which were attributed to the tissue origins related to B and T cells. The highest LCK levels were observed in THYM (thymoma), as thymus tissue was expected to have enriched T cells. Among the rest non-hematologic and non-lymphoid cancer types, we found that both the CD20 and LCK protein levels were high in the stomach adenocarcinoma (STAD). The adrenocortical carcinoma (ACC) had the lowest LCK levels, and the cervical squamous cell carcinoma and endocervical adenocarcinoma (CESC) had the lowest CD20 levels. Brain tumors, the LGG and GBM, had the second-to-third lowest LCK levels and moderate CD20 levels. These results showed the discordant infiltration of TIL-T and TIL-B cells (**Figure 3B**).

### Prognostic Significance of TIL-T and TIL-B Cells Across Different Cancer Types

**Figure 4:**
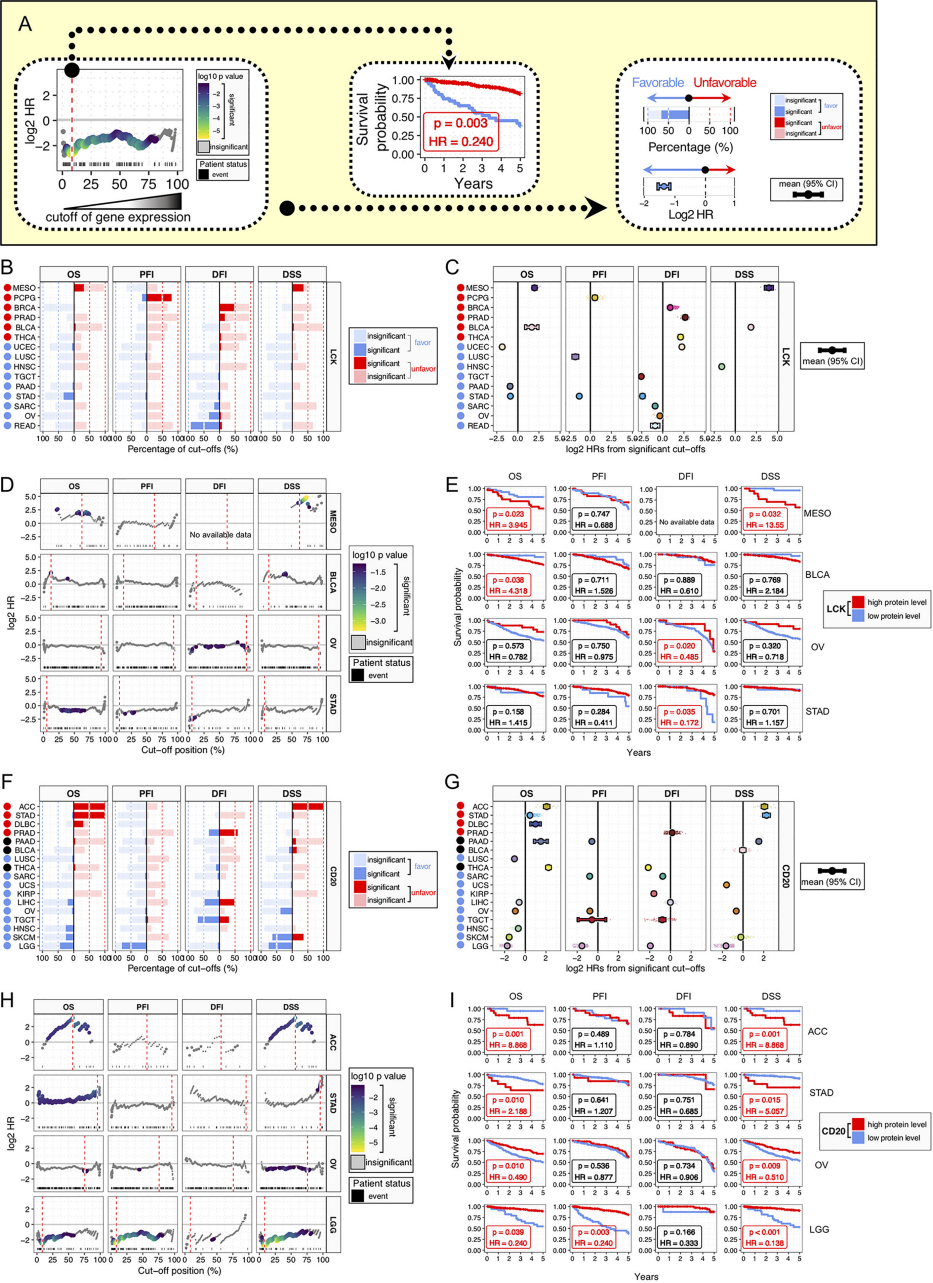
Prognostic significance of LCK and CD20 across cancer types. **(A)** Analysis framework of prognostic relevance. Patients were separated into two groups according to all the possible cut-offs of protein levels. Each subgroup should have at least 15 patients. Multivariate Cox proportional-hazards regression analysis was performed for each possible cut-off, with covariates including age, gender, histological grade, and stage. **(B, F)** Bar plots of the percentages of favorable and unfavorable cut-offs by the protein levels of LCK **(B)** and CD20 **(F)** across different cancer types and four clinical outcomes of OS, PFI, DFI, and DSS. **(C, G)** Distributions of HRs for significant cut-offs. Error bars show mean ± 95% CI. **(D, H)** Plots of HRs for individual cut-offs by the protein levels of LCK **(D)** and CD20 **(H)**, with color indicating *p*-value. The best cut-off is indicated by red dashed line. **(E, I)** Kaplan-Meier plots for patient stratification using the best cut-off of LCK **(E)** and CD20 **(I)**.

To comprehensively evaluate the prognostic value of the continuous TILs gradient, we employed an exhaustive survival analysis approach (**Figure 4A**), which calculates the significance for every possible expression cutoff exhaustively, as previously reported (22, 24, 25). Patients were stratified by all the possible cut-offs. At each cut-off, we conducted multivariate Cox proportional-hazards survival analysis, with adjustment for gender, age, histological grade, and stage. Clinical outcomes were measured by 5-year survival, using four clinical outcome endpoints of OS (overall survival), PFI (progression-free interval), DFI (disease-free survival), and DSS (disease-specific survival). Results of exhaustive survival analysis for LCK were summarized by the proportions of favorable and unfavorable cut-offs (**Figure 4B**) and the distributions of hazard ratios (HRs) for significant cut-offs (**Figure 4C**). The high LCK protein levels were associated with unfavorable prognosis in the cancer types of MESO, PCPG, BRCA, PRAD, BLCA, and THCA, while the favorable associations were found in the cancer types of READ, OV, SARC, STAD, PAAD, TGCT, HNSC, LUSC, and UCEC.

Next, we selected four cancer types with prognostic significances and plotted HRs for the continuous cut-offs (**Figure 4D**). The best cut-offs, the cut-off with the highest value of absolute log2 HR, were used for Kaplan Meier plots (**Figure 4E**). Different cancer types did not have overlapped cut-off positions in the percentage scale. Moreover, for individual cancer types, survival analyses of different endpoints showed different results, even using the best cut-off.

For the CD20, we also observed both the favorable and unfavorable associations in different cancer types (**Figures 4F–I**). The high CD20 protein levels showed unfavorable associations in the cancer types of ACC, STAD, DLBC, and PRAD, and favorable associations were found in the cancer types of LGG, SKCM, HNSC, TGCT, OV, LIHC, KIRP, UCS, SARC, and LUSC, while PAAD, BLCA, and THCA showed unclear biases towards the favorable or unfavorable outcome (**Figures 4F, G**). Next, ACC, STAD, OV, and LGG were selected for HR plots and Kaplan Meier plots (**Figures 4H, I**). The prognostic associations were notably robust, albeit with discordant biases towards the favorable or unfavorable effect. Moreover, it is noteworthy that ACC and LGG tumors had the first-to-second lowest T cell infiltrations (**Figures 3A, B**), implying that TIL-B cells could exert significant prognostic effects when only minimal TIL-T cells were present in the TME.

Collectively, both TIL-T and TIL-B cells showed heterogeneous prognostic relations in different cancer types, indicating the complex role of TILs in cancer prognosis. These results might be due to the complex interplay between the TILs and the TME. Thus, our further analysis should focus on the relationships between the TILs and TME.

### Distinct Distributions of TIL-T and TIL-B Cells in Six Subtypes of Tumor Microenvironment

**Figure 5:**
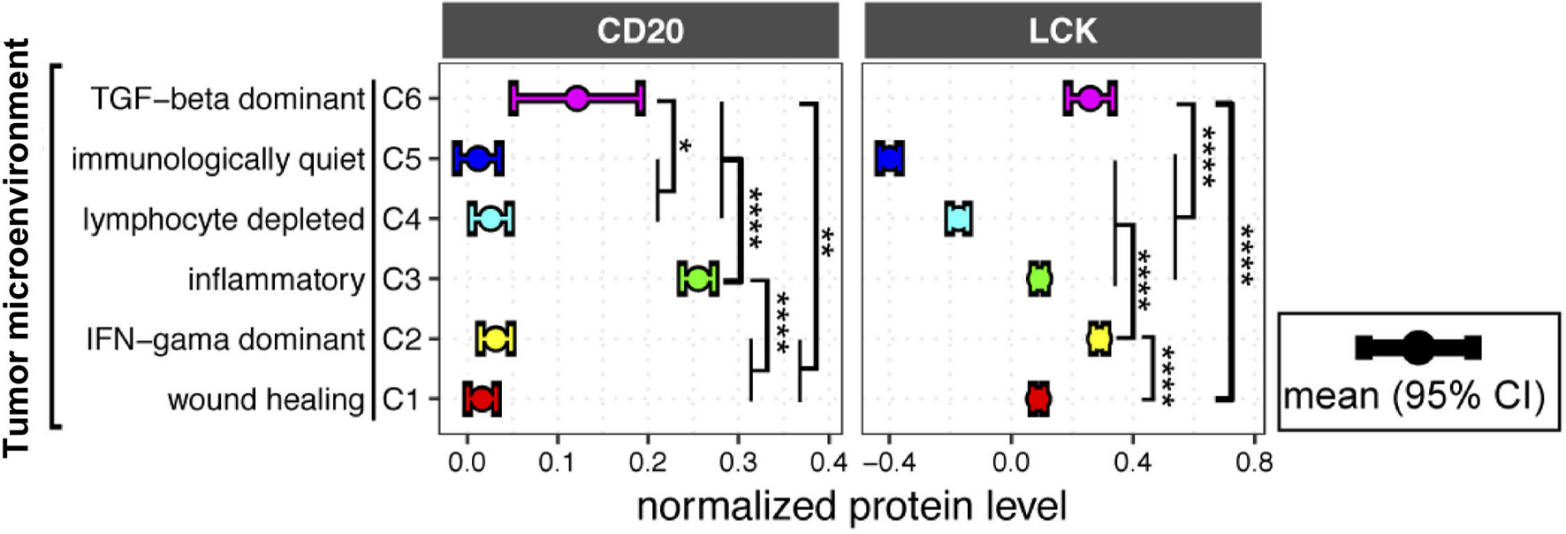
LCK and CD20 protein levels across different tumor microenvironment (TME) subtypes. CD20 and LCK protein levels in different TME subtypes. The error bar represents the 95% confidence interval (CI), the dot represents the average value, with color coding according to cancer type. The p-value was calculated using the ANOVA test (*p-value<0.05, **p-value<0.01, ***p-value<0.001, ****p-value<0.0001).

The previous study has identified six immune subtypes of TME, which have been defined as wound healing, IFN-γ dominant, inflammatory, lymphocyte depleted, immunologically quiet, and TGF-β dominant signatures (1). Here, we found that the immunological quiet subtype had the lowest LCK and CD20 protein levels, and the lymphocyte depleted subtype also had very low LCK and CD20 protein levels (**Figure 5**), indicating the concordant deletion of TIL-T and TIL-B cells. Notably, both the protein levels of CD20 and LCK were the second-highest in the TGF-β dominant subtype (**Figure 5**), which was the most minority subgroup with only 180 cases that composed only 2.0% of the total cases. The TGF-β dominant subtype has the highest TGF-β expression, which might contribute to the concordant enrichment of TIL-T and TIL-B cells. Of note, we observed that the wound healing subtype, defined by elevated expression of angiogenic genes, had moderate LCK levels but very low CD20 levels. The inflammatory subtype, the TME subtype with the highest mRNA levels of Th17 genes, had the prominently highest CD20 protein levels and moderate LCK protein levels. And, the IFN-γ dominant subtype had the prominently highest LCK levels but very low CD20 levels (**Figure 5**). The above results showed that the distributions of TIL-B and TIL-T cells were not fully concordant across different TME subtypes.

### Distinct Distributions of TIL-T and TIL-B Cells in Subgroups of Gender, Histopathology, and Histological Grade

Next, we analyzed the CD20 and LCK protein levels in different subgroups defined by gender (**Figure S9**), histopathology (**Figure S10**), and histological grade (**Figure S11**). In the gender-related differences, males had significantly lower LCK protein levels than females in the lung squamous cell carcinoma (LUSC), breast invasive carcinoma (BRCA), and bladder urothelial carcinoma (BLCA), and males had significantly higher CD20 protein levels in the LUSC, esophageal carcinoma (ESCA) and glioblastoma multiforme (GBM) (**Figure S9**).

In the histopathology-related differences, the adenocarcinoma had significantly higher LCK protein levels than the squamous cell carcinoma in lung cancer and ESCA. And, adenocarcinoma had significantly lower CD20 protein levels in lung cancer (**Figure S10**).

In the histological-grade-related differences, the higher grade tumor had significantly higher LCK protein levels than the lower grade tumor in STAD, kidney renal clear cell carcinoma (KIRC), liver hepatocellular carcinoma (LIHC), and BLCA; and the higher grade tumor had significantly lower CD20 protein levels in the ovarian serous cystadenocarcinoma (OV) and BLCA but significantly higher CD20 levels in STAD (**Figure S11**).

The above results revealed the distinct distributions of TILs in different subgroups, suggesting the importance of analyzing the prognostic relevance of TILs in different subgroups separately.

### The Prognostic Landscape of TIL-B and TIL-T Cells Across Different Cancer Types and the Clinical, Therapeutic, and TME Subgroups

**Figure 6:**
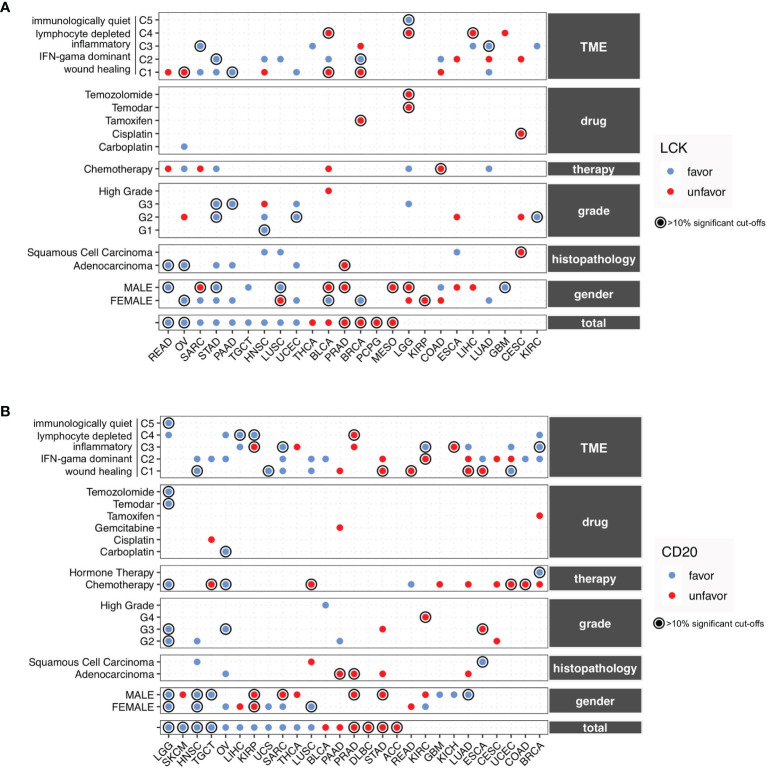
The prognostic landscape of CD20 and LCK in different cancer types and subgroups defined by gender, histopathology, histopathological grade, therapeutic approach, drug, and TME. **(A, B)** Prognostic significances of CD20 **(A)** and LCK **(B)** in the total cancer types and different subgroups, with favorable and unfavorable associations indicated by blue and red dots. The prognostic significances were shown using the average percentage of the significant cut-offs from OS, PFI, DFI, and DSS if available. The robust associations with percentages of more than 10% were underlined with circles. The detailed results of subgroup analyses could be found in **Figures S12–17**.

Previous studies have reported the prognostic associations of TIL-T and TIL-B cells in several cancer types (11–14). However, there is a scarcity of data on the prognostic landscape of TIL-B and TIL-T cells in heterogeneous cancer subgroups. To solve the problem, we conducted subanalyses based on the clinical, therapeutic, and TME subtypes. The TCGA cohorts of 32 cancer types were separated into different subgroups by gender, histopathology, histological grade, therapeutic approach, drug, and TME subtypes. All the subgroups were used for conducting the exhaustive multivariate Cox proportional-hazards regression. The detailed results of the subgroup analyses were provided in the supplementary information (**Figures S12–17**). Moreover, we presented the summary plots that include the significant prognostic relevance of LCK and CD20 for individual cancer types and subgroups (**Figures 6A, B**). Of these, we underlined the robust prognostic significances that have more than 10% significant cut-offs.

For the TIL-T cells measured by LCK, the favorable associations were observed in STAD, PRAD, TGCT, and UCEC, and the unfavorable associations were found in PRAD, MESO, KIRP, and CESC, whereas the rest cancer types showed inconsistent prognostic relevance (**Figure 6A**). For the TIL-B cells measured by CD20, the favorable associations were observed in LGG, HNSC, OV, and UCS, and the unfavorable associations were found in PRAD, DLBC, STAD, and ACC, while the other cancer types showed inconsistent prognostic relevance in different subgroups (**Figure 6B**).

Of note, the TIL-B and TIL-T cells, measured by the CD20 and LCK protein levels, showed different prognostic trends across various subgroups, which indicated that the TILs play a complex role under the heterogeneous context of cancer subtypes. Therefore, the discordant effects of TIL-T and TIL-B cells might not solely be driven by the heterogeneous clinical, therapeutic, and TME subtypes.

### Potential Modulators of T-Cell Infiltration Across Different Cancer Types

The TGF-β dominant TME subtypes have high levels of TIL-T cells (**Figure S18A**). Next, to identify the specific source of TGF-β at the cellular level, we analyzed the expression of TGFB1, TGFB2, and TGFB3 using the sc-RNAseq datasets of 33K PBMC and 52K lung cancer cells (21). The expression levels of TGFB2 and TGFB3 were extremely low, while the TGFB1 expressed dominantly in the monocytes in blood and the macrophages in cancer tissues (**Figures S18B–C**). The IFN-γ dominant subtype displayed the highest IFN-γ expression signature, which is the most dominant TME subtype, which composed 28.4% of the total cases. The previous study of IFN-γ dominant TME has analyzed the IFN-γ dominant communication network, including CXCL9, CXCL10, CXCR3, CCL5, IFNG, IFNGR1, and IFNGR2 (1). Based on the IFN-γ dominant communication network, we inferred the cellular context for the communications between TME and TILs. Through analyzing the expression of well-acknowledgeable receptor-ligand pairs using the sc-RNAseq datasets, we combined the IFN-γ dominant TME communication network with the cell-type-specific knowledge at the single-cell level.

Here, we found that CXCL9 and CXCL10, the IFN-γ inducible chemokines (26), were rarely expressed in the monocytes derived from blood (**Figure S18D**). However, macrophages in lung cancer tissues expressed high levels of CXCL9 and CXCL10 (**Figure S18E**). CXC chemokine receptor 3 (CXCR3), the receptor for CXCL9 and CXCL10 (26), was expressed dominantly in T and NK cells from both peripheral blood and lung cancer (**Figures S18D, E**). These findings suggested that NK cells and T cells in the blood might migrate into the tumor tissues due to the communications between the macrophages and the T cells. Next, the NK and T cells also dominantly expressed IFNG and CCL5, a potent chemoattractant whose acknowledgeable function is to recruit monocytes and macrophages (27). IFNGR1 and IFNGR2, the cognate receptors of IFNG, were expressed in both monocytes in blood and macrophages in lung cancer (**Figures S18D, E**).

**Figure 7:**
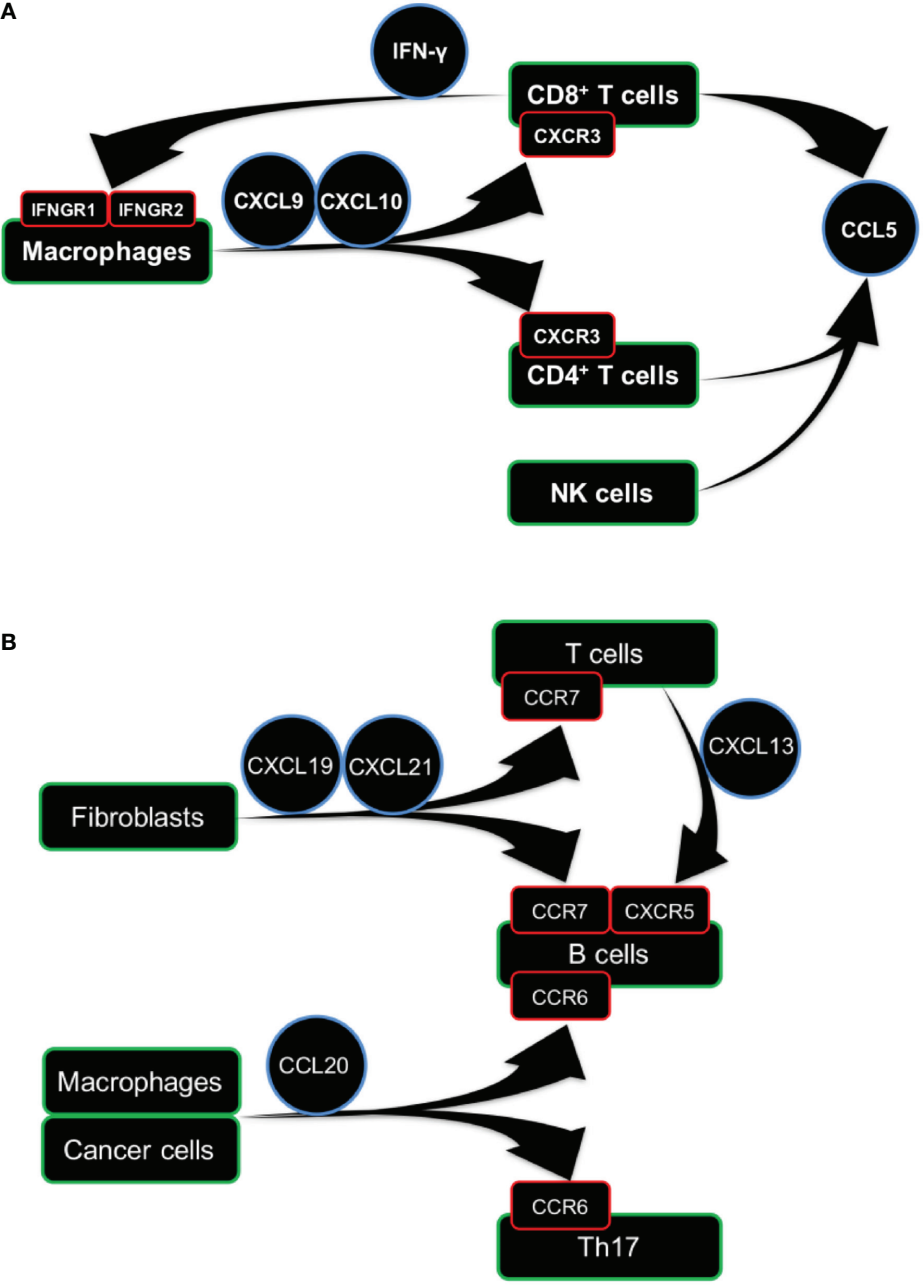
Cellular communication network involving TIL-T and TIL-B cells. **(A, B)** Graphical representation of cellular communication network of TIL-T **(A)** and TIL-B **(B)** cells. Arrow represents the directions of ligand-receptor pairs expressed in the corresponding cells.

The TIL-T cells expressed IFNG, CCL5, and CXCR3 that play the central role in the IFN-γ dominant communication network (**Figure S18F**). Next, we analyzed the relationships of LCK with IFNG, CCL5, and CXCR3, using TCGA bulk RNA-sequencing data, and we found significant associations in the expression levels of LCK with IFNG (**Figure S18G**, total rho=0.73), CCL5 (**Figure S18H**, total rho=0.80), and CXCR3 (**Figure S18I**, total rho=0.84) in most cancer types. The above findings were illustrated by the graphical representation (**Figure 7A**), indicating the cross-talks between macrophages and T cells involved the IFN-γ dominant communication network.

It is reported that TIL-T cells express immune checkpoint genes, such as programmed cell death protein 1(PDCD1, PD-1) and the cytotoxic T lymphocyte-associated protein 4 (CTLA-4), which renders TIL-T ineffective against tumors (6, 8). Recent immunotherapies have used PD-1, CTLA4, and TIGIT inhibitors to enhance T cell response in cancer patients (6–8). Here, we found that the PD-1 (PDCD1) and CTLA4 were only expressed in a sub-cluster of T cells in the peripheral blood (**Figures S19A–C**), but the vast majority of TIL-T cells in lung cancer tissues expressed PD-1 and CTLA4 (**Figure S19D**). Next, the TIGIT, an T cell exhaustion marker and inhibitory receptor for both NK and T cells (7), was expressed dominantly on both T and NK cells. And, the TIGIT-expressing T cells were largely overlapped with the PD-1 and CTLA4-expressing T cells (**Figures S19A–D**). Furthermore, we found that LCK was significantly correlated with TIGIT (**Figure S19E**, total rho=0.82), CTLA4 (**Figure S19F**, total rho=0.77), and PD-1 (**Figure S19G**, total rho=0.81) in most cancer types, indicating the important role of TIGIT, CTLA4, and PD-1 in the TIL-T cells across cancer types.

### Potential Modulators of B-Cell Infiltration Across Different Cancer Types

The TIL-B cells were remarkably enriched in the inflammatory TME (**Figure 5**). The inflammatory TME subtype is defined by the signature of high expression of the Th17 gene (1). Here, we analyzed the CCR6 and CXCR4, the important homing molecules expressed by Th17 (28). And, it is reported that the CCR6 was consistently expressed in both Th17 and B cells across blood, lymphoid, and non-lymphoid tissues (29). Using the sc-RNAseq datasets, we found that the CCR6 was highly expressed in both B cells and a sub-cluster of T cells in both blood and lung cancer tissues (**Figures S20A, B**). However, the CXCR4 was expressed in almost all the cell subsets in both blood and lung cancer tissues, indicating that CXCR4 was not a specific marker for Th17. Next, we analyzed the ligands of CCR6 and CXCR4, which are the CCL20 (30) and CXCL12 (31). The CCL20 was expressed in cancer cells and macrophages in lung cancer tissues, while the CXCL12 was expressed by fibroblasts in lung cancer tissues (**Figures S20A, B**). These findings indicated that the migration and retention of both B and Th17 cells in tumor tissue might be controlled by the communications between the B cells, Th17 cells, macrophages, and cancer cells through the CCR6-CCL20 and CXCR4-CXCL12 chemokine axes.

Next, we analyzed CCR7, the homing molecule expressed by B cells (32) and CD8^+^ T cells (33) but not expressed by Th17 (34). Here, we found that the CCR7 was expressed by the B and T cells in both blood and lung cancer tissues. And, the CCL19 and CCL21, the ligands of CCR7 (35, 36), were expressed by the fibroblasts in lung cancer tissues (**Figures S20A, B**). These results indicated that both B and T cells could migrate into tumor tissues from peripheral blood, which might depend on the communications with fibroblasts through the CCR7-CCL19/CCL21 chemokine axis.

Next, we analyzed the CXCR5, which has been reported to be co-expressed with CCR6 and CCR7 in B cells (29). We found that the CXCR5 was expressed in B cells from both PBMCs and lung cancer. And, the CXCL13, the ligand for CXCR5 (37), was expressed in T cells from the lung cancer tissues (**Figures S20A, B**). These findings implied that the B cells in blood could be recruited to tumor tissue by the TIL-T cells through the CXCR5-CXCL13 chemokine axis (**Figure S20C**).

Next, we observed significant associations of the expression levels of CD20 with CCR6 (**Figure S20D**, total rho=0.62), CCR7 (**Figure S20E**, total rho=0.79), and CXCR5 (**Figure S20F**, total rho=0.73) in most cancer types. The above findings indicated that the CCR6, CCR7, and CXCR5, expressed in the B cells, might play an important role in the infiltration of TIL-B cells across different cancer types. Based on the above results, we constructed the cellular communication network for TIL-B cells (**Figure 7B**), which also showed the infiltrations of T and B cells might involve different but partially overlapped molecular interaction networks.

## Discussion

Previously, the heterogeneous TME was classified into six distinct subtypes through different signatures of the immune gene expression (1). Additionally, these six TME subtypes were characterized by distinct distributions of leukocyte fractions, which were estimated using the DNA methylation probes with the greatest differences between pure leukocyte cells and normal tissue. Moreover, the spatial fractions of lymphocyte regions, assessed by digitized H&E-stained slides, were also different in these TME subtypes (3). In our study, we provided the protein-level measurements of the TIL-T and TIL-B abundances, which added a novel dimension for the evaluation of TILs. We observed distinct TIL-T and TIL-B distributions across different TME subtypes, which is in line with the previous characterizations of TILs in those TME subtypes. Notably, we found that the enrichments of TIL-T and TIL-B cells were not entirely consistent, indicating the involvement of different cellular communication networks.

Through mapping the well-acknowledgeable receptor-ligand pairs on the cellular context at the single-cell level, we inferred the cellular communication network involving the TIL-T or TIL-B cells. We found the crosstalk between the TIL-T cells and macrophages and the communications of TIL-B cells with cancer cells, macrophages, fibroblasts, and TIL-T cells. Consistent with our expectations, these results indicate the TME regulations of TIL-T and TIL-B cells through the complex but partially overlapped communication network. Notably, despite these complicated TME interactions and heterogeneous TME subtypes, the communication network highlights several conserved chemokine receptors on TIL-T and TIL-B, respectively. These chemokine receptors showed remarkably and positively relations with the TIL-T and TIL-B abundances across various cancer types. These findings could be accounted for by assuming that, although the TILs communication network may differ under heterogeneous TME contexts, the respective key regulators of TIL-T and TIL-B abundances are likely to be consistent across different cancer types.

In our study, we observed varying distributions of TIL-T and TIL-B cell abundances across diverse cancer types and subgroups. Furthermore, in different cancer types and subgroups, both TIL-T and TIL-B cells showed heterogeneous prognostic effects. It is reported that the heterogeneous prognostic effects of the TIL-T and TIL-B cells could probably be attributed to the heterogeneous constitution of TIL-T and TIL-B subpopulations (38–40). Different TIL-T and TIL-B cell subsets have distinct functions, which may contribute to pro- and antitumorigenic responses. Here, our study reports the prognostic landscape of TIL-T and TIL-B cells, which offered a preliminary indicator for the pro- and antitumorigenic roles of TIL-T and TIL-B cells in different cancer types and subgroups as well as response to different therapy and drugs.

To date, the immunotherapy of manipulating TILs has become a critical treatment for cancer patients (6–8). Thus, our study might have important implications for immunotherapy. Recently, the antitumorigenic effect of CD8^+^ TIL-T cells has been extensively investigated and used in immunotherapy. However, only part of CD8^+^ TIL-T cells was specific for tumor antigen (41). In contrast, another subgroup of CD8^+^ TIL-T cells was characterized as bystander T cells that recognize a wide range of epitopes unrelated to cancer (41). In this study, the abundances of bulk TIL-T cells showed adverse prognostic effects in different cancer types. Thus, our knowledge of TIL-T cells is far from complete. The antigenic specificity of TIL-T cells should also be considered in future studies.

In comparison with TIL-T cells, the TIL-B cells are less well studied. In our study, we found that the TIL-B cells showed remarkable prognostic relevance. It is still unclear whether TIL-B cells reflect or play an important role in the specific immune responses to tumors. Future studies could help to study the function of TIL-B cells in the prognostic-related cancer types, such as ACC, STAD, OV, and LGG. Also, the pro- and antitumorigenic TIL-B cell subpopulations remain to be determined. With a better understanding of TIL-B cells, it could be feasible to design immunotherapies that target not only TIL-T cells but also the TIL-B cells to improve cancer patients’ survival.

Our study has several advantages over previous studies. Firstly, we used standardized, normalized, and batch corrected LCK and CD20 protein levels across 32 cancer types, which allowed us to investigate the distributions of TIL-T and TIL-B abundances spanning different tumor types. Moreover, we demonstrated that tumors from different tissues had a remarkable difference in the abundances of lymphocytic context. Secondly, we performed an exhaustive multivariate survival analysis considering all the possible cut-offs, which reduced the potential for erroneous conclusions drawn from a single cut-off. Previously, the survival analysis usually used the median-split approach that equally divides patients into two groups. However, the median-split approach has a major limitation that real-world data inevitably have distributional variations, which is often ignored. For instance, for the DFI of CD20 in LIHC, we observed 50.82% favorable and 49.18% significant unfavorable cutoffs (**Figure 4F**), which indicates that the prognostic significance could be confounded using a single arbitrary cut-off. Comparatively, the exhaustive multivariate survival analysis could measure the prognostic relationship more reliably. Moreover, the percentage of the significant cut-offs could be used to represent the robustness of the prognostic relationship.

Through the improved methodology, we found that, in the LGG patients, the TLL-T cells showed insignificant prognostic associations, which were presented as 0% significant cutoffs. In contrast, the high TIL-B cell abundances were remarkably associated with favorable prognosis, and the percentages of significant favorable cutoffs were 43.95%, 79.01%, 5.319%, and 75.69% for OS, PFI, DFI, and DSS, respectively (**Figure 4F**). Additionally, the favorable role of TIL-B cells was also observed in different LGG subgroups. To the best of our knowledge, it is the first time to identify the prognostic significance of TIL-B cells in LGG cancer patients. Although further researches are required involving independent patient cohorts, the weight of evidence strongly supports a positive role for TIL-B cells in LGG, suggesting that enhancing rather than inhibiting TIL-B cell responses might be considered for the design of immunotherapies for LGG patients.

There are still some limitations in our study. Firstly, although our study used 5-year survival, the TCGA cohorts have different follow-up times across different cancer types (17). Secondly, those TCGA cohorts also have different survival event rates (17). Thirdly, the OS, PFI, DFI, and DSS are different definitions of clinical outcomes in oncology research, and as described in the recommendation of using the data of TCGA clinical outcomes, the OS and PFI could be relatively accurate, the DFI is reasonably accurate, but DSS could only be estimated for most cases (17). It remains unknown how much the above limitations might bias our results across different cancer types and subgroups. Thus, we recommend that those prognostic associations with less than 10% significant cut-offs should be interpreted cautiously. Nevertheless, most of our survival analyses have clear prognostic relevance towards the favorable or unfavorable trend. Notably, prognostic associations with more than 10% significant cut-offs could be preferentially used for further study.

Taken together, to the best of our knowledge, this study depicted the first high-resolution prognostic landscape of TIL-T and TIL-B cell abundances across heterogeneous human malignancies and the clinical, therapeutic, and TME subgroups. The prognostic landscape indicates lots of hypotheses for future study, including exploring the function of TILs under different clinical and TME subtypes or elucidating the effects of TILs on different therapeutic approaches and drugs. Despite these hypothetical possibilities, the precise functions of TIL-T and TIL-B cells in the TME require further study. Nevertheless, this study comprehensively investigated the impact of TIL-T and TIL-B cells on cancer patients’ survival across different cancer types, the prognostic landscape of TIL-T and TIL-B cells will be a useful resource for future studies seeking to better understand the role of TILs in cancer subtypes in which it has not been explored, with critical implications for cancer immunotherapy.

## Data Availability Statement

The TCGA data can be found at the legacy archive of the GDC (https://portal.gdc.cancer.gov/). Details for data availability are in the *Materials and Methods*.

## Ethics Statement

No human subjects were directly involved in this study. All the data used in this study was derived from existing de-identified biological samples from prior studies. Therefore, ethical and patient consent was not required in this study.

## Author Contributions

Concept and design: MZ. Development of the methodology: MZ. Acquisition of the data: MZ, Y-ML, Z-YL, XZ, and XY. Analysis and interpretation of the data: MZ. Preparation, review, and/or revision of the manuscript and figures: MZ and YZ. Study supervision: MZ, J-LJ, JT, Z-NC, and PZ. All authors read and approved the final manuscript.

## Funding

This work was supported by grants from the National Natural Science Foundation of China (32100739) and the National Science and Technology Major Projects of New Drugs (2014ZX09508002-002).

## Conflict of Interest

The authors declare that the research was conducted in the absence of any commercial or financial relationships that could be construed as a potential conflict of interest.

## Publisher’s Note

All claims expressed in this article are solely those of the authors and do not necessarily represent those of their affiliated organizations, or those of the publisher, the editors and the reviewers. Any product that may be evaluated in this article, or claim that may be made by its manufacturer, is not guaranteed or endorsed by the publisher.

## Supplementary Material

Figure S1 t-SNE projection of single-cell RNA-sequencing data from 28,823 human PBMCs, with each dot representing one single cell and colors representing the expression of known canonical marker genes **(A)** and 5 major cell lineages **(B)**.

Figure S2 **(A–B)** Comparisons between the factions of NK cells and T cells across 25 cancer types in 7,741 ImmunePRECOG samples. Error bars showing mean ± 95% confidence interval (CI). The p-value was calculated using the Student’s t-test (**p*-value<0.05, ***p*-value<0.01, ****p*-value<0.001, *****p*-value<0.0001).

Figure S3 **(A–B)** Comparisons between the factions of NK cells vs T cells across 33 cancer types in 11,273 TCGA samples. Error bars showing mean ± 95% confidence interval (CI). The p-value was calculated using the Student’s t-test (**p*-value<0.05, ***p*-value<0.01, ****p*-value<0.001, *****p*-value<0.0001).

Figure S4 The t-SNE projection of single-cell RNA-sequencing data from 7,865 human PBMCs (10K PBMC dataset), with colors representing the canonical marker genes’ mRNA level **(A)**, protein level **(B)**, and major cell lineages **(C–D)**.

Figure S5 The t-SNE projection of single-cell RNA-sequencing data from 52,698 human lung cancer cells (52K lung cancer dataset), with red color representing different cell lineages.

Figure S6 The relationships between the expression levels of CD3E and LCK, CD19, and CD20 across different cancer types. **(A–B)** Plots showing co-expression between LCK and CD3E **(A)**, CD20 and CD19 **(B)** across different cancer types. Statistics were computed using Spearman’s rank correlation.

Figure S7 The relationships between the mRNA and protein levels of CD20 and LCK in cell lines from different tissue sources. Statistics were computed using Spearman’s rank correlation.

Figure S8 Bar plot of sample sizes in different cancer types from the TCGA RPPA dataset.

Figure S9 CD20 and LCK protein expression in different cancer types stratified by gender. The p-value was calculated using the Student’s t-test (**p*-value<0.05, ***p*-value<0.01, ****p*-value<0.001, *****p*-value<0.0001).

Figure S10 CD20 and LCK protein expression in different cancer types stratified by histopathology. The p-value was calculated using the Student’s t-test (**p*-value<0.05, ***p*-value<0.01, ****p*-value<0.001, *****p*-value<0.0001).

Figure S11 CD20 and LCK protein expression in different cancer types stratified by histological grade. The p-value was calculated using the ANOVA (**p*-value<0.05, ***p*-value<0.01, ****p*-value<0.001, *****p*-value<0.0001).

Figure S12 Subgroup survival analysis for gender. Statistics of survival analysis were computed using multivariate Cox proportional hazards survival analysis using age, histological grade, and stage if applicable. **(A, C)** Bar plots of the percentages of favorable and unfavorable cut-offs by the protein levels of CD20 **(A)** and LCK **(C)** across 4 clinical outcomes, including OS, PFI, DFI and DSS and TCGA cancer types subgroup by gender. **(B, D)** HRs of significant cut-offs. Error bars show mean ± 95% CI.

Figure S13 Subgroup survival analysis for histopathology. Statistics of survival analysis were computed using multivariate Cox proportional hazards survival analysis using gender, age, histological grade, and stage if applicable. **(A, C)** Bar plots of the percentages of favorable and unfavorable cut-offs by the protein levels of CD20 **(A)** and LCK **(C)** across 4 clinical outcomes, including OS, PFI, DFI and DSS and TCGA cancer types subgroup by histopathology. **(B, D)** HRs of significant cut-offs. Error bars show mean ± 95% CI.

Figure S14 Subgroup survival analysis for histological grade. Statistics of survival analysis were computed using multivariate Cox proportional hazards survival analysis using gender, age, and stage if applicable. **(A, C)** Bar plots of the percentages of favorable and unfavorable cut-offs by the protein levels of CD20 **(A)** and LCK **(C)** across 4 clinical outcomes, including OS, PFI, DFI and DSS and TCGA cancer types subgroup by histological grade. **(B, D)** HRs of significant cut-offs. Error bars show mean ± 95% CI.

Figure S15 Subgroup survival analysis for therapeutic approach. Statistics of survival analysis were computed using multivariate Cox proportional hazards survival analysis using gender, age, histological grade, and stage if applicable. **(A, C)** Bar plots of the percentages of favorable and unfavorable cut-offs by the protein levels of CD20 **(A)** and LCK **(C)** across 4 clinical outcomes, including OS, PFI, DFI and DSS and TCGA cancer types subgroup by therapeutic approach. **(B, D)** HRs of significant cut-offs. Error bars show mean ± 95% CI.

Figure S16 Subgroup survival analysis for drug. Statistics of survival analysis were computed using multivariate Cox proportional hazards survival analysis using gender, age, histological grade, and stage if applicable. **(A, C)** Bar plots of the percentages of favorable and unfavorable cut-offs by the protein levels of CD20 **(A)** and LCK **(C)** across 4 clinical outcomes, including OS, PFI, DFI and DSS and TCGA cancer types subgroup by drug. **(B, D)** HRs of significant cut-offs. Error bars show mean ± 95% CI.

Figure S17 Subgroup survival analysis for tumor microenvironment subtype. Statistics of survival analysis were computed using multivariate Cox proportional hazards survival analysis using gender, age, histological grade, and stage if applicable. **(A, C)** Bar plots of the percentages of favorable and unfavorable cut-offs by the protein levels of CD20 **(A)** and LCK **(C)** across 4 clinical outcomes, including OS, PFI, DFI and DSS and TCGA cancer types subgroup by immune subtype. **(B, D)** HRs of significant cut-offs. Error bars show mean ± 95% CI.

Figure S18 CD20 and LCK protein levels in different subtypes of tumor microenvironment (TME), and the cellular communications involving IFN-γ dominant communication network. **(A)** CD20 and LCK protein levels in different TME subtypes. **(B–E)** Plots of t-SNE projections for 33K PBMC and 52K lung cancer sc-RNAseq datasets, with color indicating the normalized expression of genes from the TGF-β family **(B–C)** and the IFN-γ dominant communication network **(D, E)**. **(F)** Graphical representation for the IFN-γ dominant communication network. **(G–I)** Plots showing co-expression of LCK with IFNG **(G)**, CCL5 **(H)**, and CXCR3 **(I)** across different cancer types. Statistics were computed using Spearman’s correlation.

Figure S19 The expression of CTLA4, PDCD1, TIGIT and the tumor-infiltrating T cells. **(A–D)** Plots of t-SNE projections of single-cell RNA-sequencing datasets of 10K PBMC **(A, B)**, 33K PBMCs **(C)**, and 52K lung cancer cells **(D)**, with color indicating the normalized expression levels of CTLA4, PDCD1, and TIGIT. **(E, F)** Plots showing co-expression of LCK with TIGIT **(E)**, CTLA4 **(F)**, and PDCD1 **(F)** across different cancer types. Statistics were computed using Spearman’s correlation.

Figure S20 Cellular communication network involved tumor-infiltrating B cells. **(A, B)** Plots of t-SNE projections for sc-RNAseq datasets of 33K PBMCs **(A)** and 52K lung cancer cells **(B)**, with color indicating the normalized expression levels of B cell chemokine receptors and their ligands. **(C)** Graphical representation of cellular communication network of tumor-infiltrating B cells. **(D–F)** Plots showing co-expression of CD20 with CCR6 **(D)**, CCR7 **(E)**, and CXCR5 **(F)** across different cancer types. Statistics were computed using Spearman’s rank correlation.
