# Supplementary material for: Prognostic Landscape of Tumor-Infiltrating T and B Cells in Human Cancer
Source: Front Immunol. 2022 Jan 4;12:731329. doi: 10.3389/fimmu.2021.731329 (PMC8771864; doi:10.3389/fimmu.2021.731329)

Supplementary Figure 12

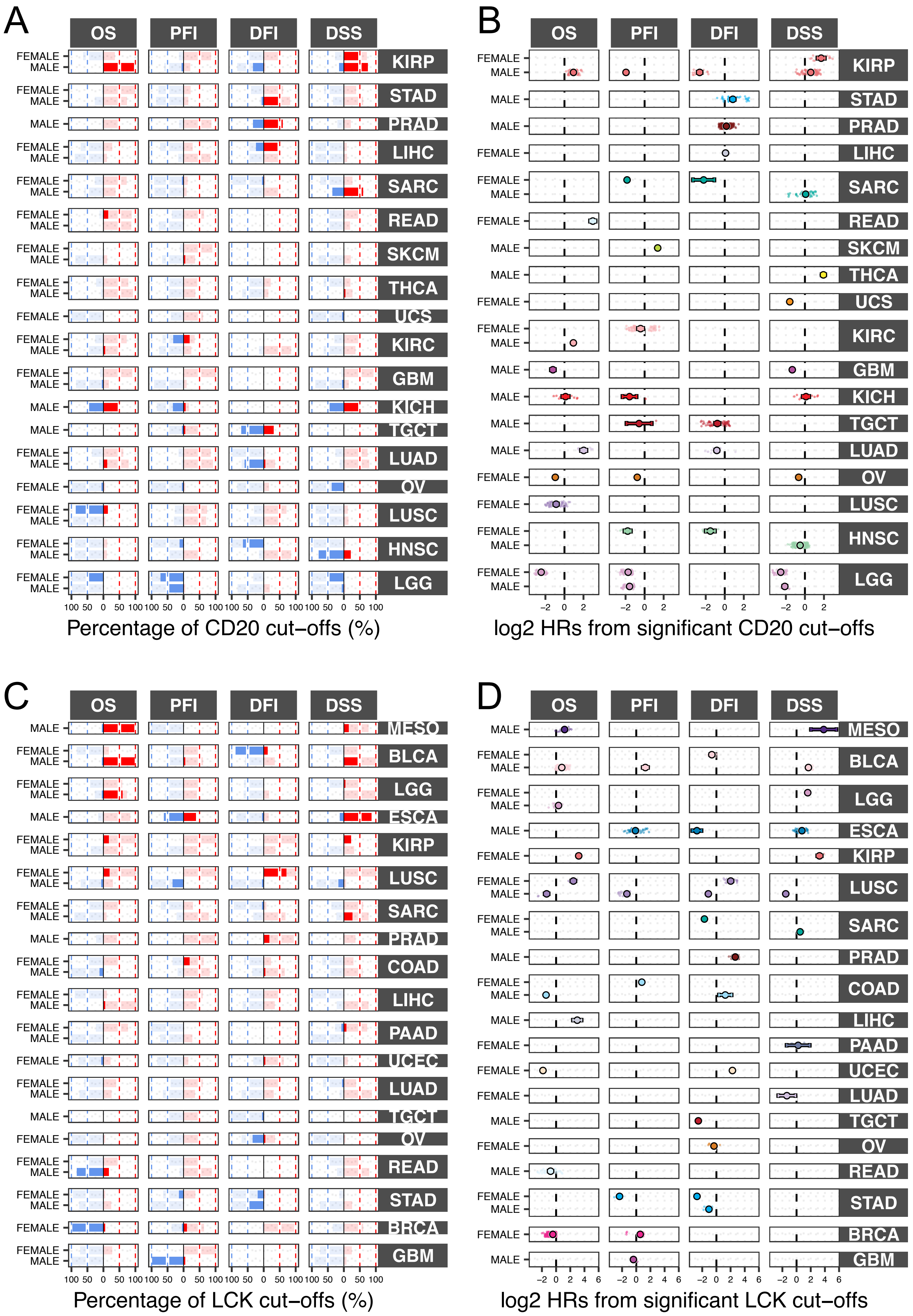

Supplementary Figure 13

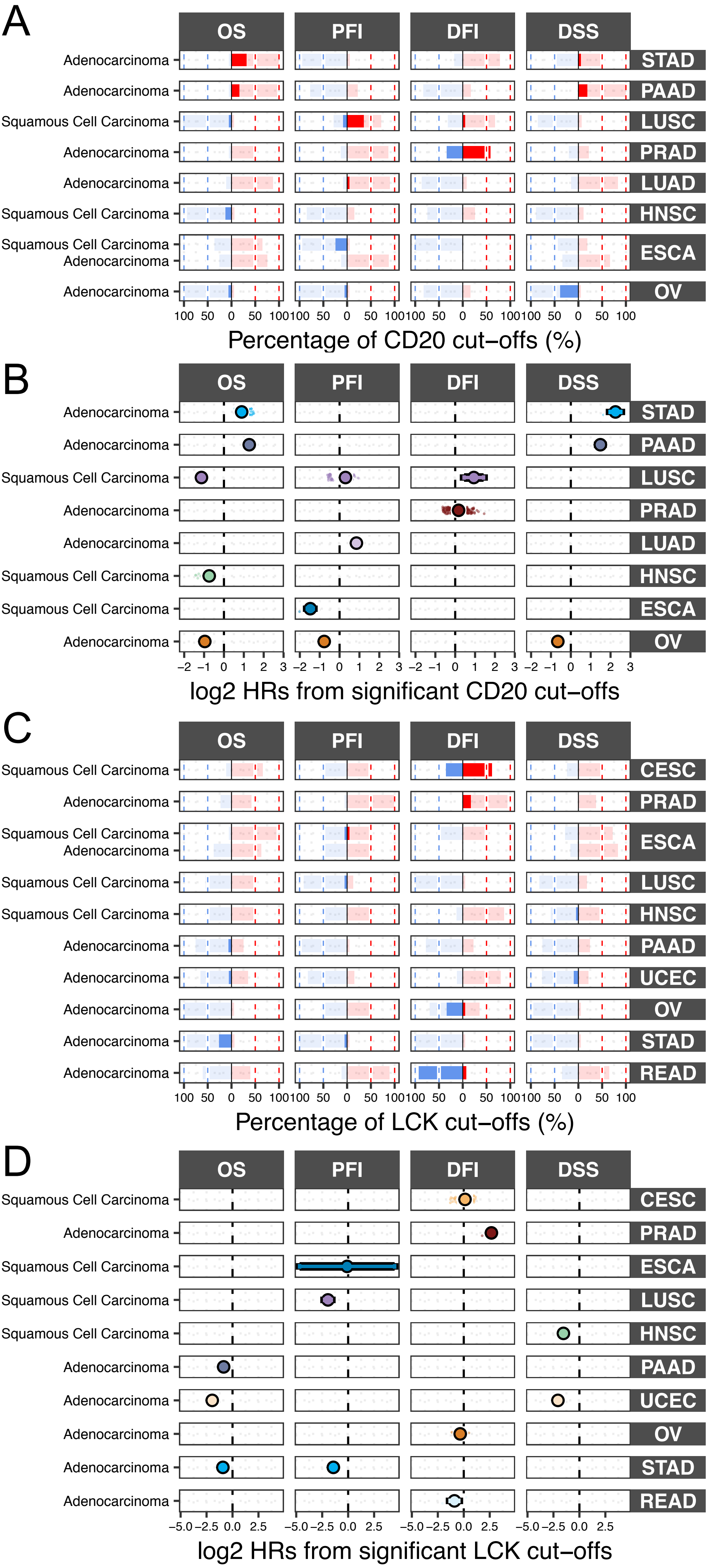

Supplementary Figure 14

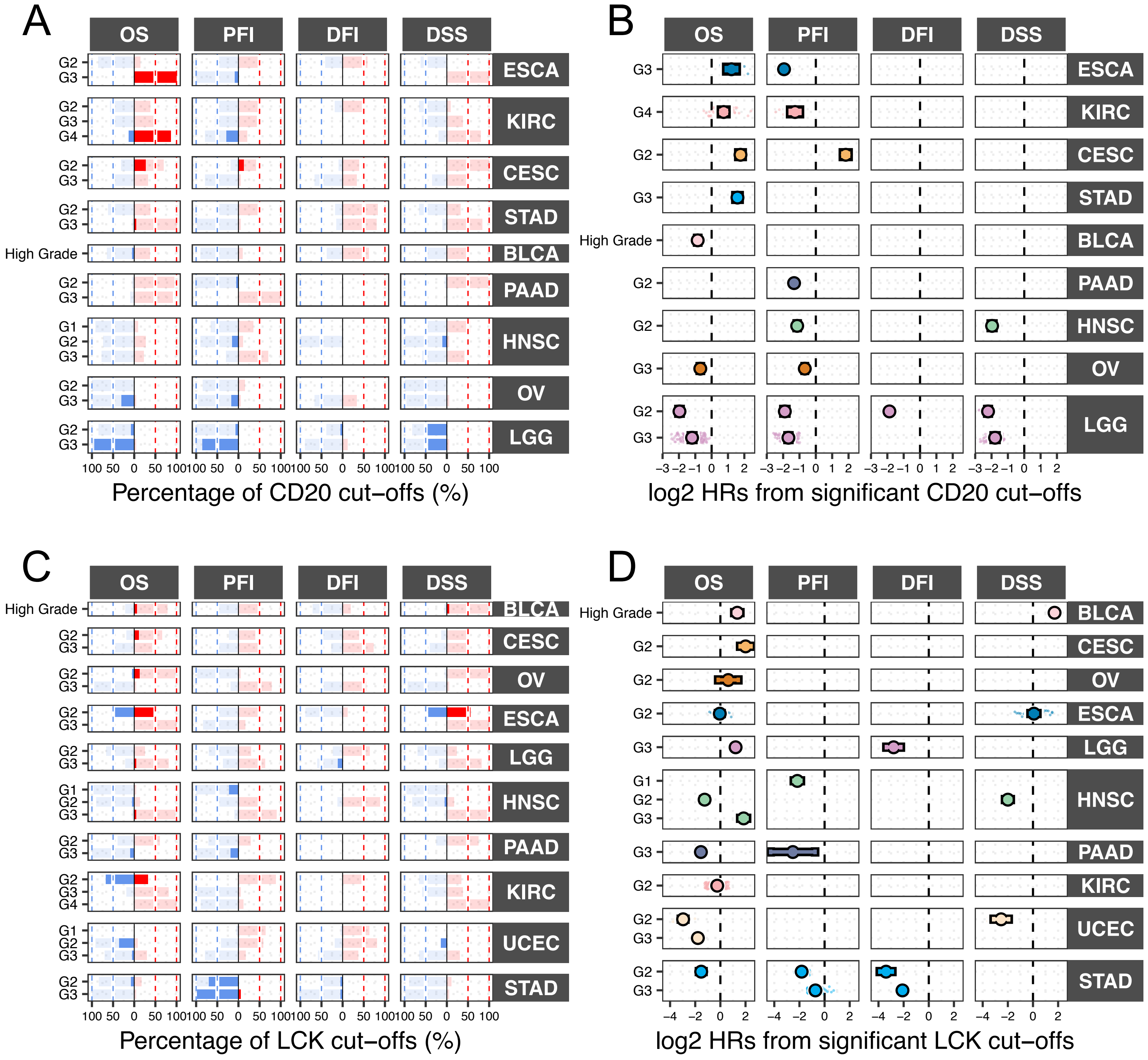

Supplementary Figure 15

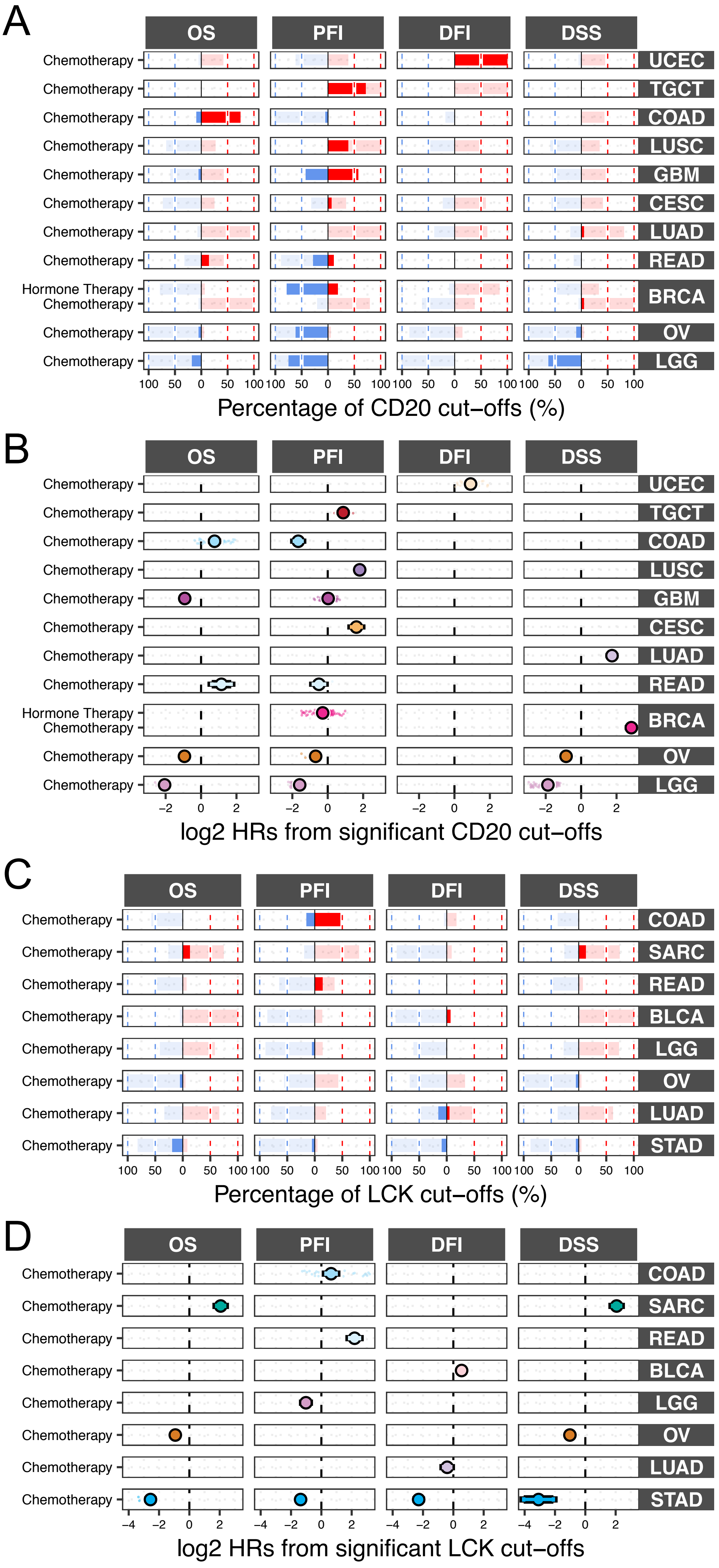

Supplementary Figure 16

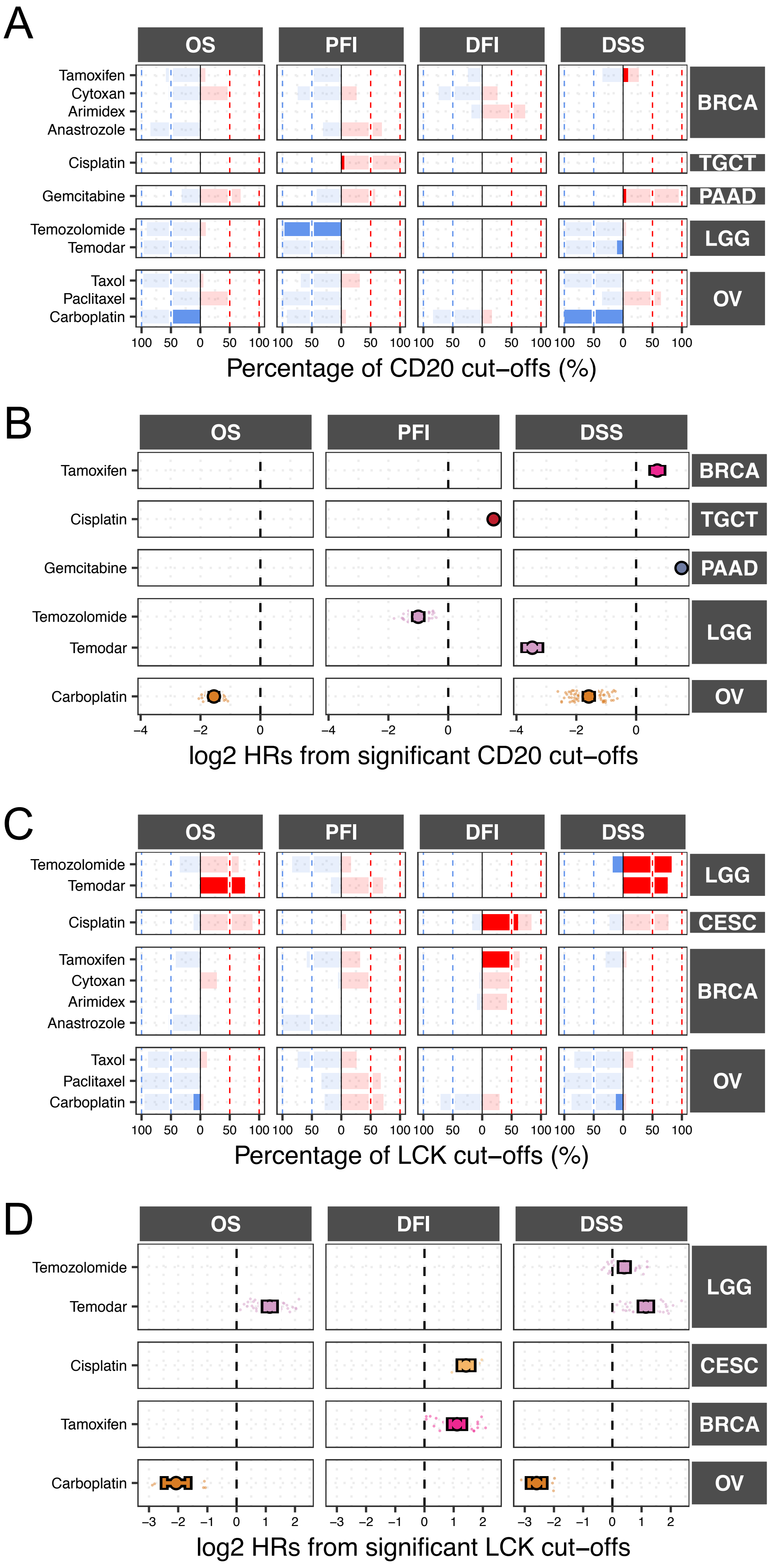

Supplementary Figure 17

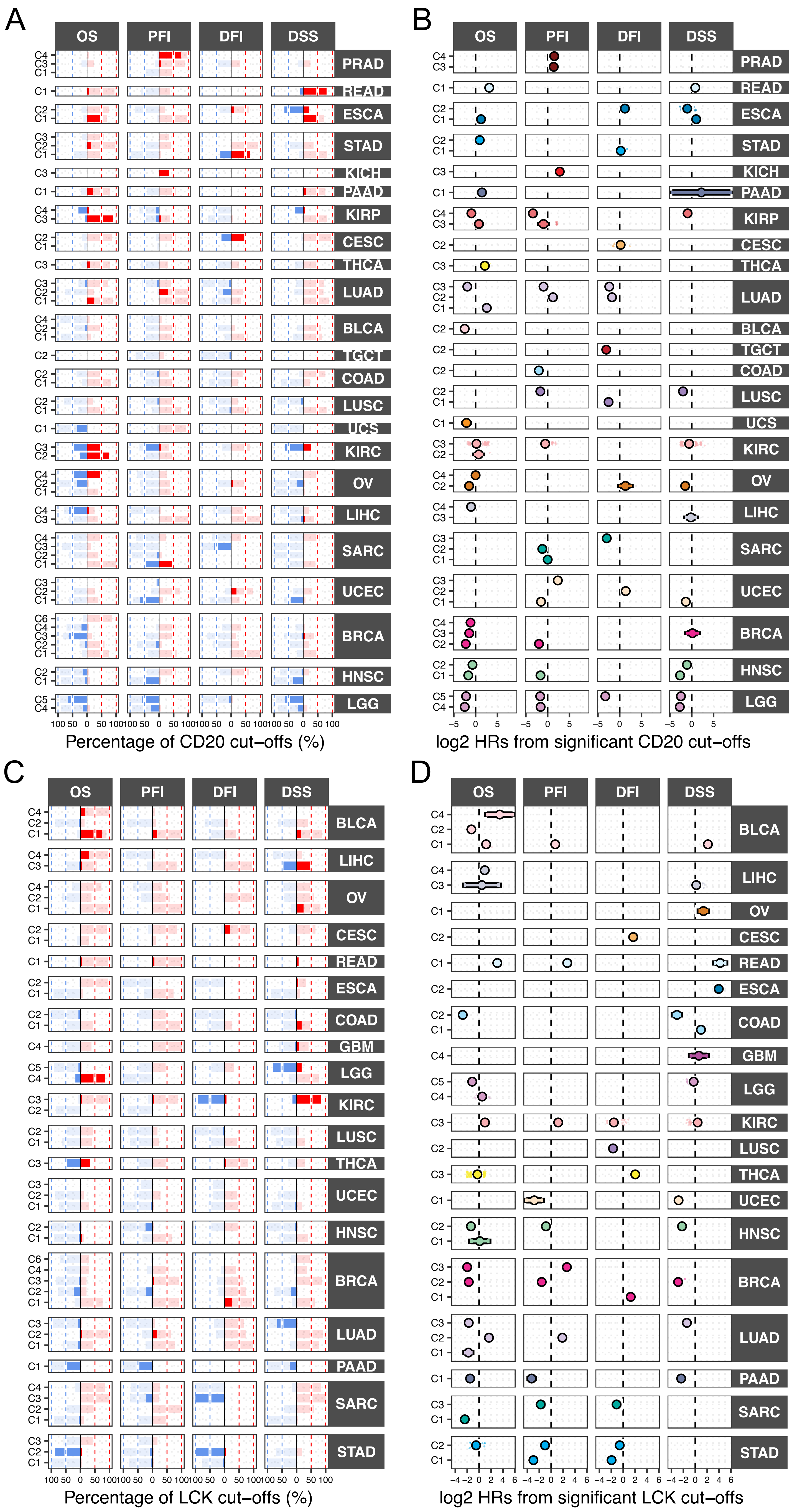

Supplement: Figure S1 — t-SNE projection of single-cell RNA-sequencing data from 28,823 human PBMCs, with each dot representing one single cell and colors representing the expression of known canonical marker genes (A) and 5 major cell lineages (B). [file DataSheet_1.zip › Supplementary_Figure_12-17.pdf]
