# Supplementary figures and images for: Prognostic Landscape of Tumor-Infiltrating T and B Cells in Human Cancer
Source: Front Immunol. 2022 Jan 4;12:731329. doi: 10.3389/fimmu.2021.731329 (PMC8771864; doi:10.3389/fimmu.2021.731329)

Supplementary Figure 9

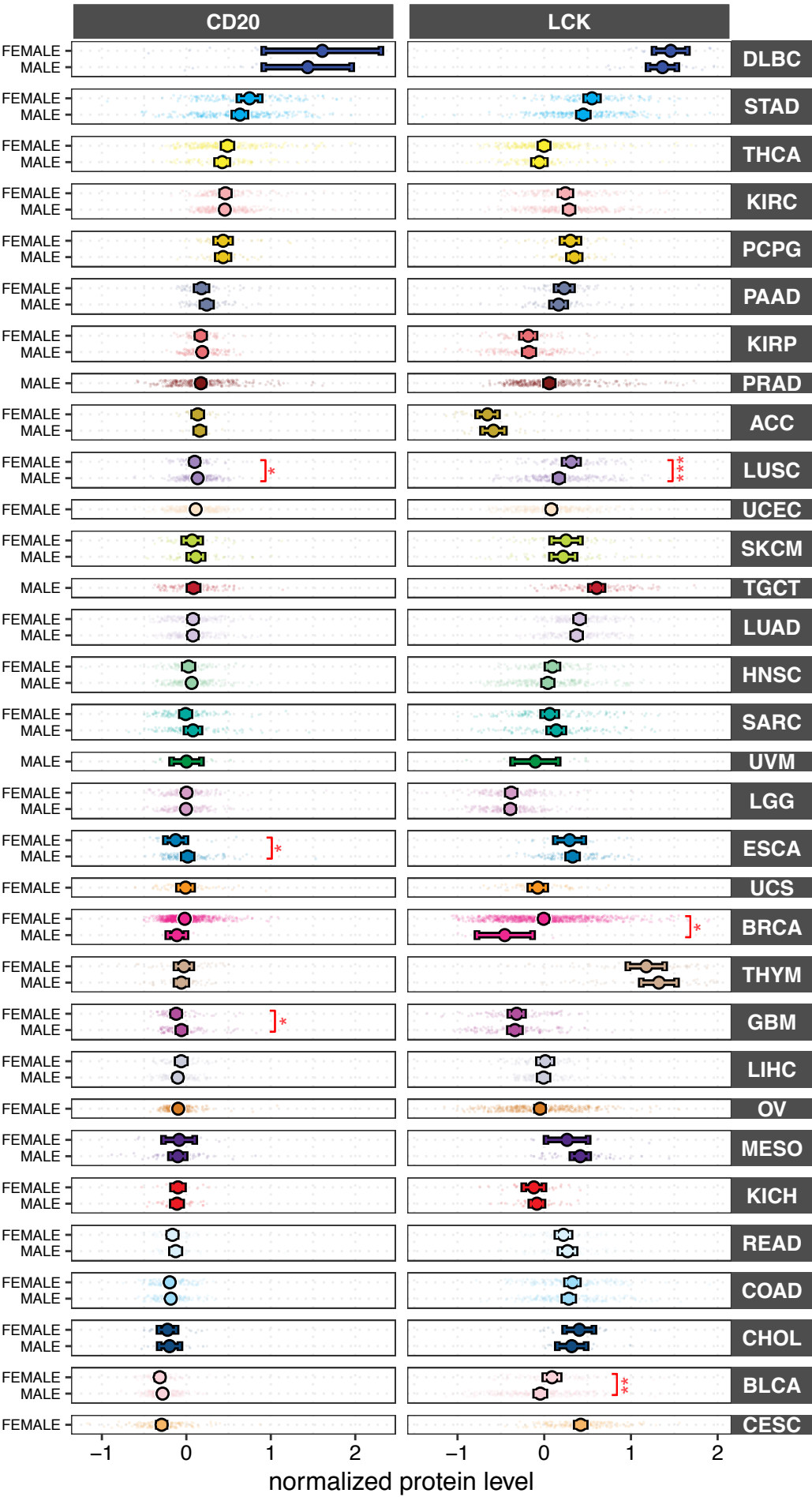

# Supplementary Figure 10

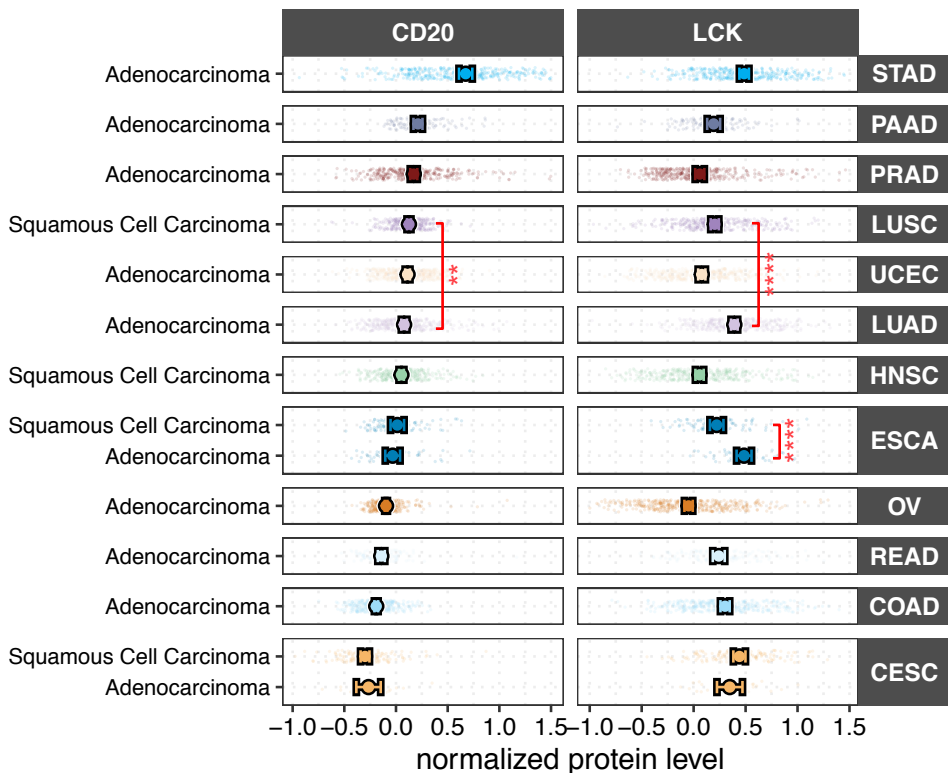

# Supplementary Figure 11

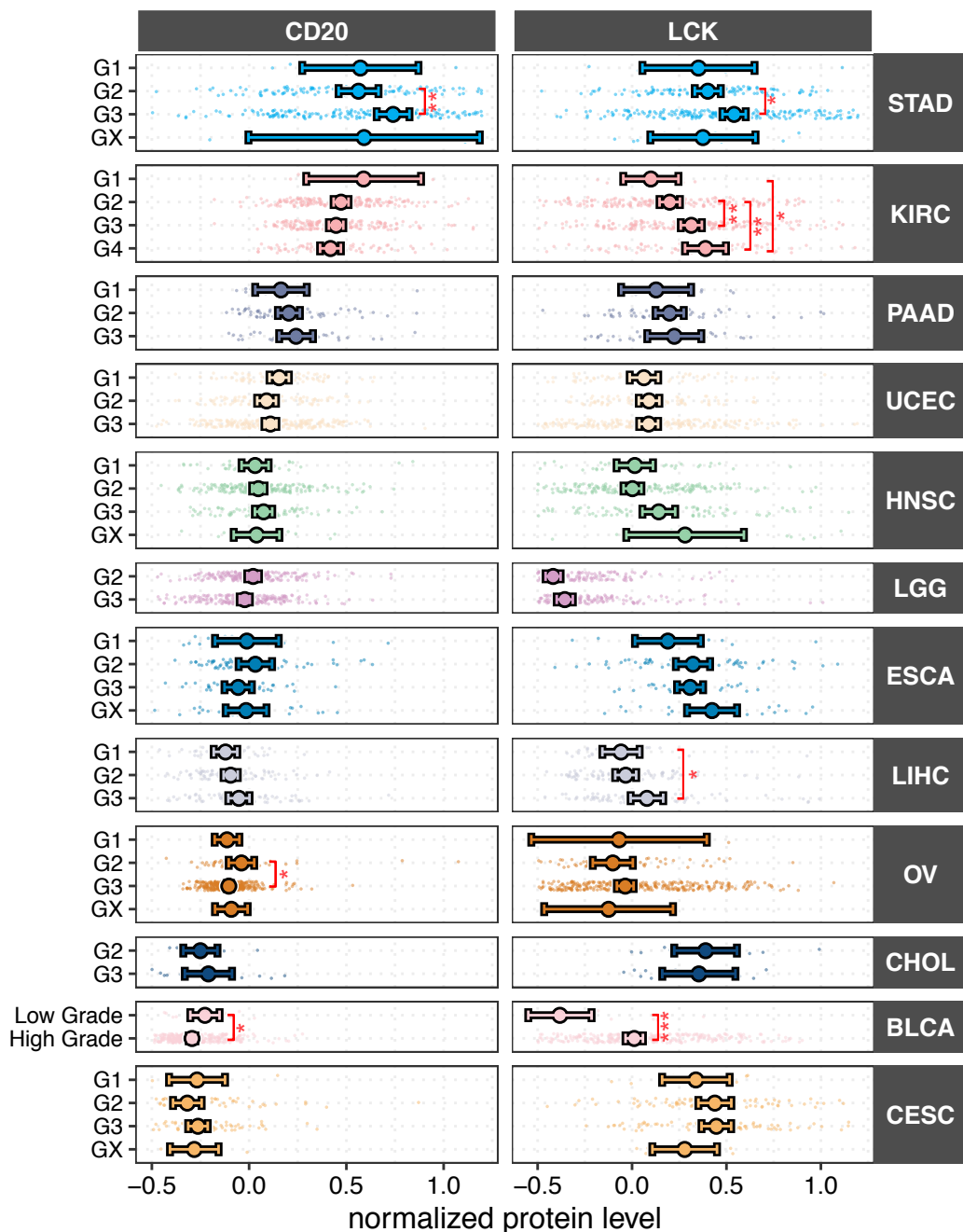

Supplement: Figure S1 — t-SNE projection of single-cell RNA-sequencing data from 28,823 human PBMCs, with each dot representing one single cell and colors representing the expression of known canonical marker genes (A) and 5 major cell lineages (B). [file DataSheet_1.zip › Supplementary_Figure_9-11.pdf]

# Supplementary Figure 20

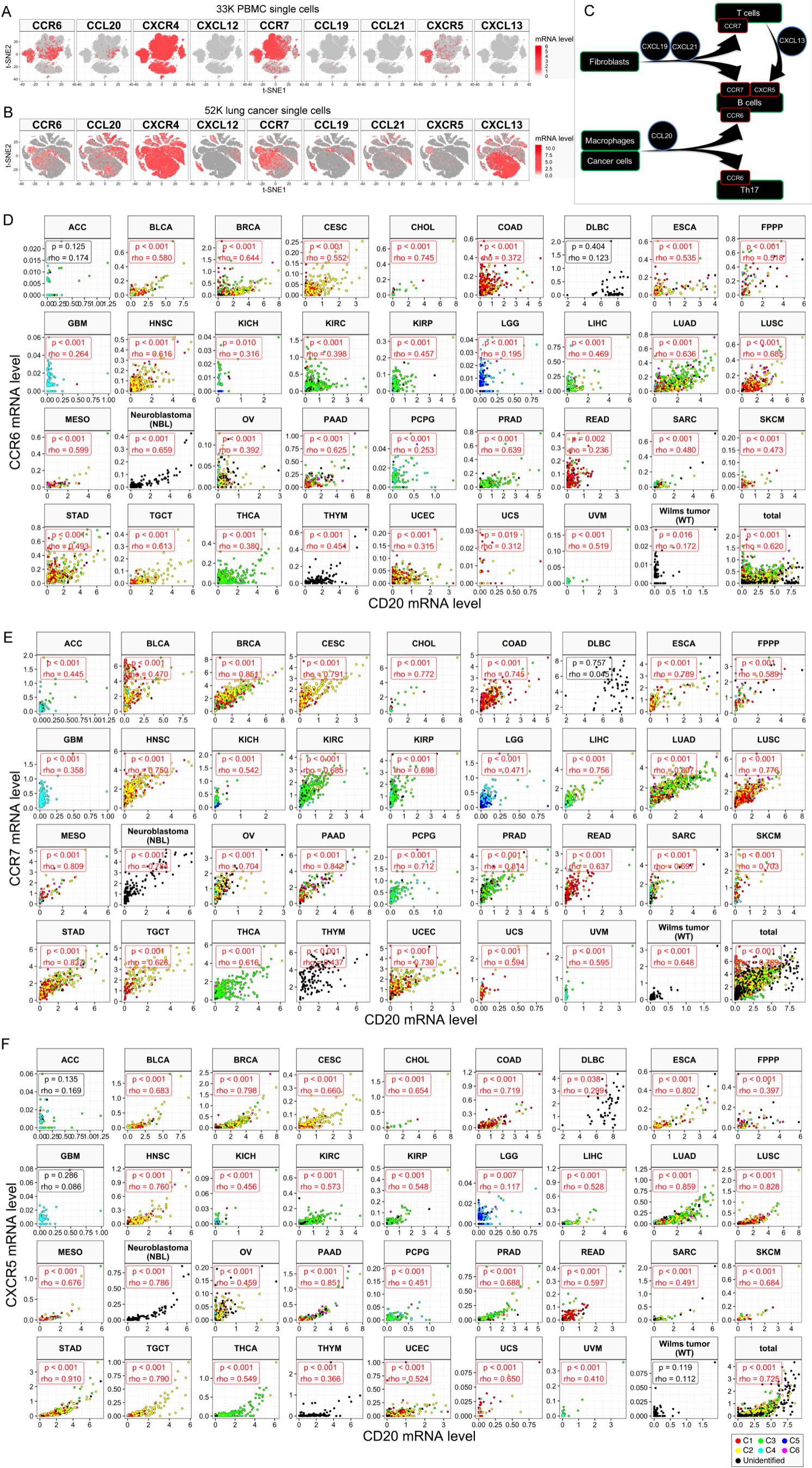

Supplement: Figure S1 — t-SNE projection of single-cell RNA-sequencing data from 28,823 human PBMCs, with each dot representing one single cell and colors representing the expression of known canonical marker genes (A) and 5 major cell lineages (B). [file DataSheet_1.zip › Supplementary_Figure_20.pdf]

# Supplementary Figure 8

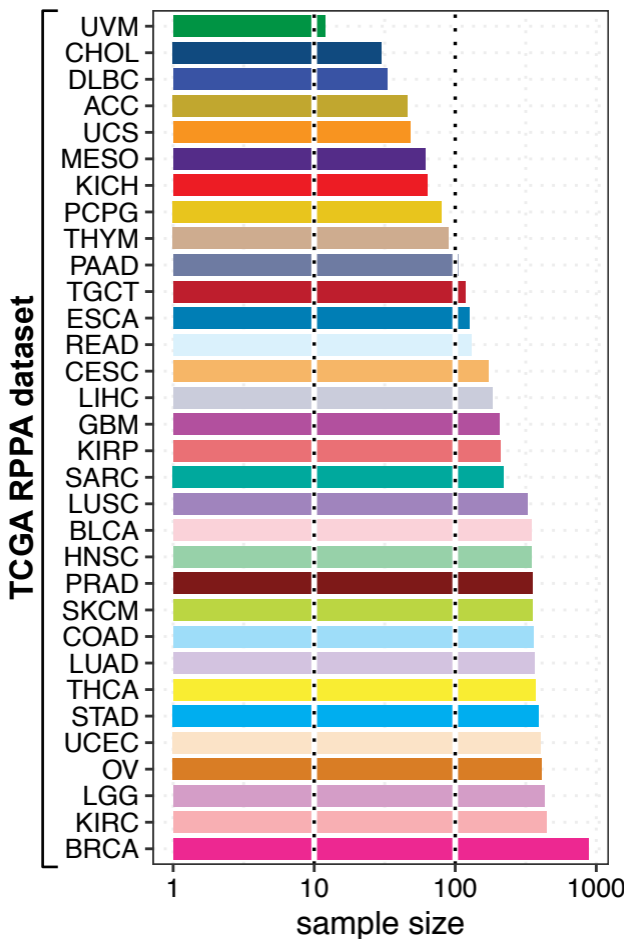

Supplement: Figure S1 — t-SNE projection of single-cell RNA-sequencing data from 28,823 human PBMCs, with each dot representing one single cell and colors representing the expression of known canonical marker genes (A) and 5 major cell lineages (B). [file DataSheet_1.zip › Supplementary_Figure_8.pdf]

Supplementary Figure 18

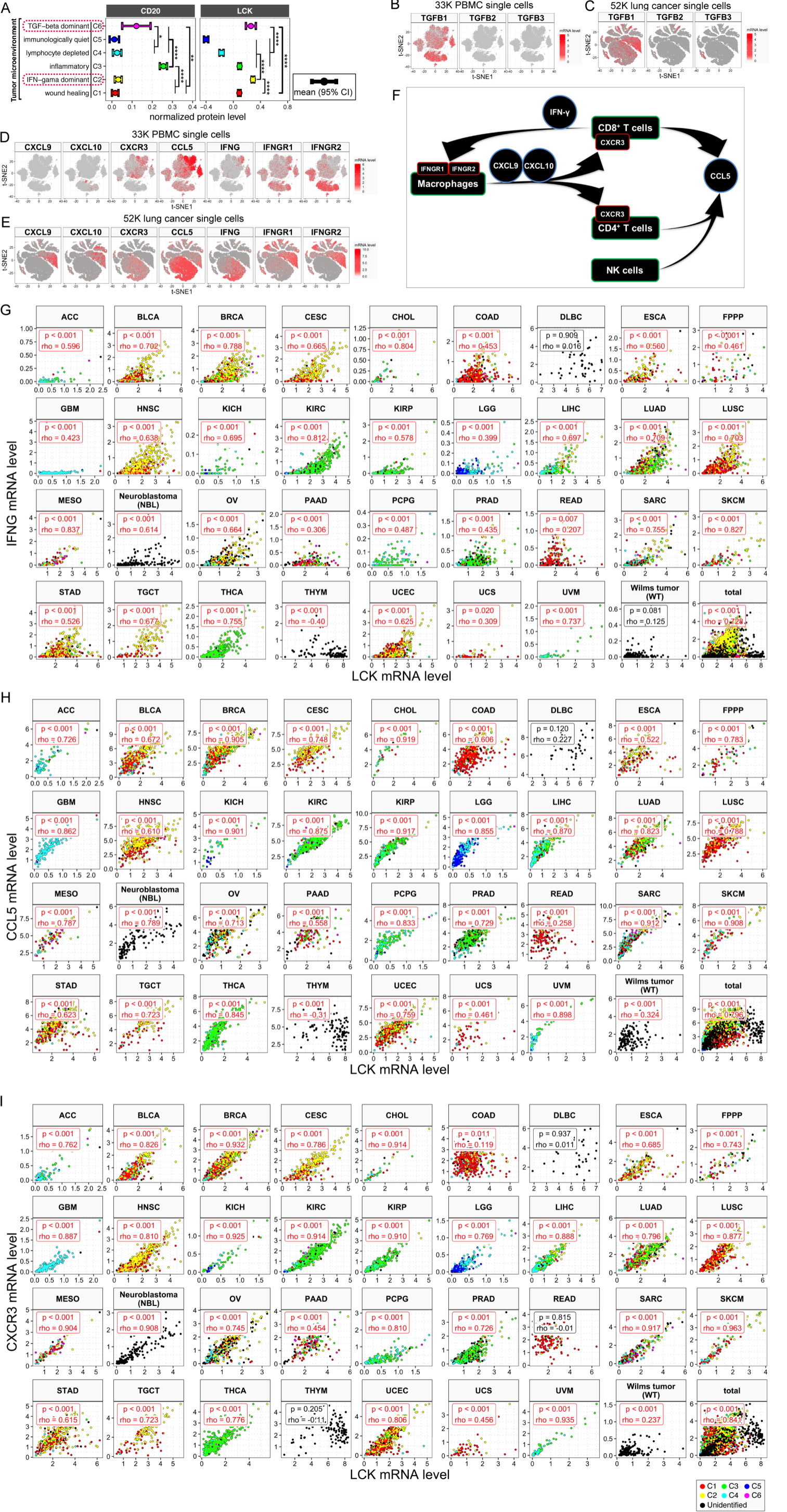

Supplement: Figure S1 — t-SNE projection of single-cell RNA-sequencing data from 28,823 human PBMCs, with each dot representing one single cell and colors representing the expression of known canonical marker genes (A) and 5 major cell lineages (B). [file DataSheet_1.zip › Supplementary_Figure_18.pdf]

# Supplementary Figure 19

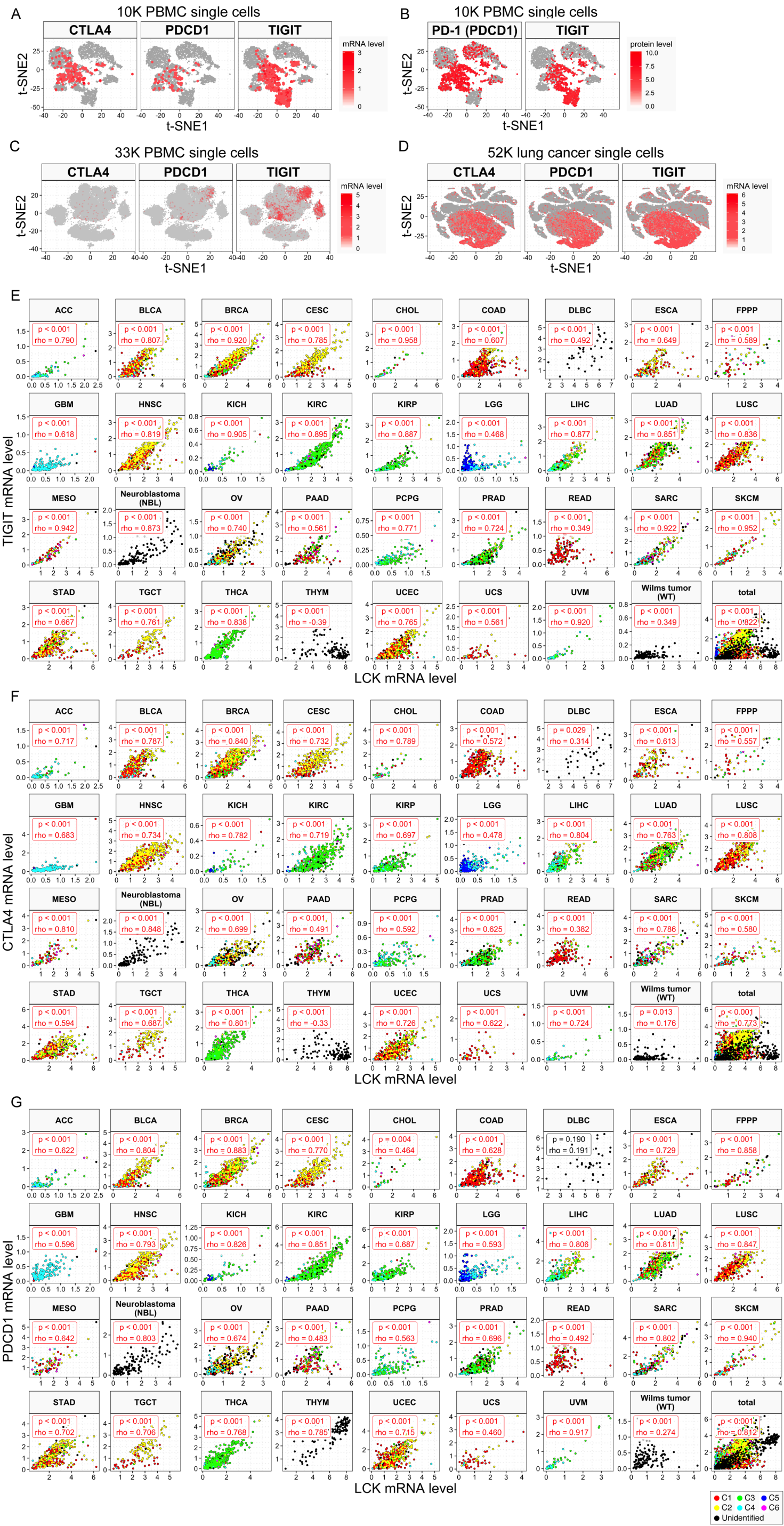

Supplement: Figure S1 — t-SNE projection of single-cell RNA-sequencing data from 28,823 human PBMCs, with each dot representing one single cell and colors representing the expression of known canonical marker genes (A) and 5 major cell lineages (B). [file DataSheet_1.zip › Supplementary_Figure_19.pdf]

# Supplementary Figure 3

A

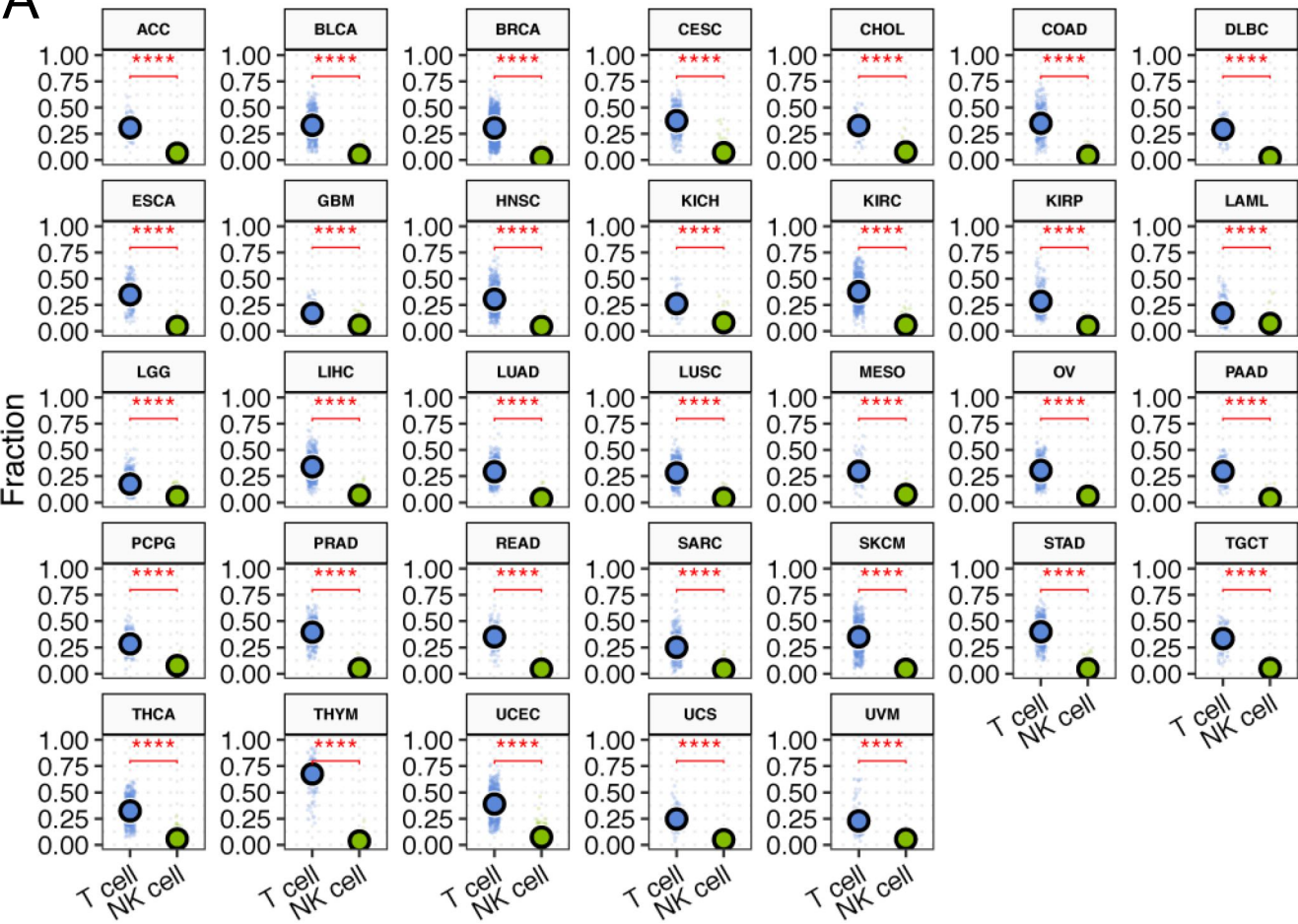

11,273 CIBERSORT TCGA samples

B

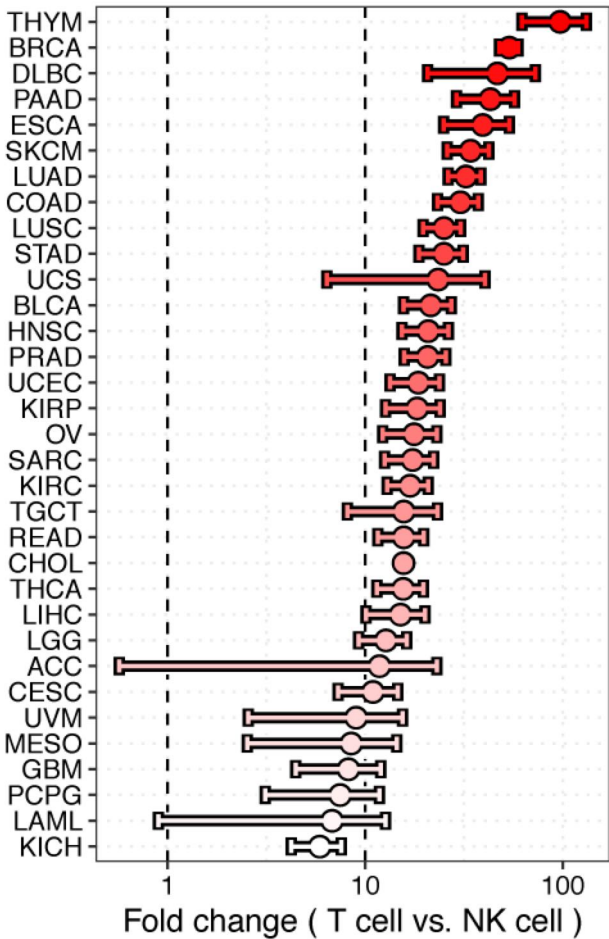

Supplement: Figure S1 — t-SNE projection of single-cell RNA-sequencing data from 28,823 human PBMCs, with each dot representing one single cell and colors representing the expression of known canonical marker genes (A) and 5 major cell lineages (B). [file DataSheet_1.zip › Supplementary_Figure_3.pdf]

Supplementary Figure 2

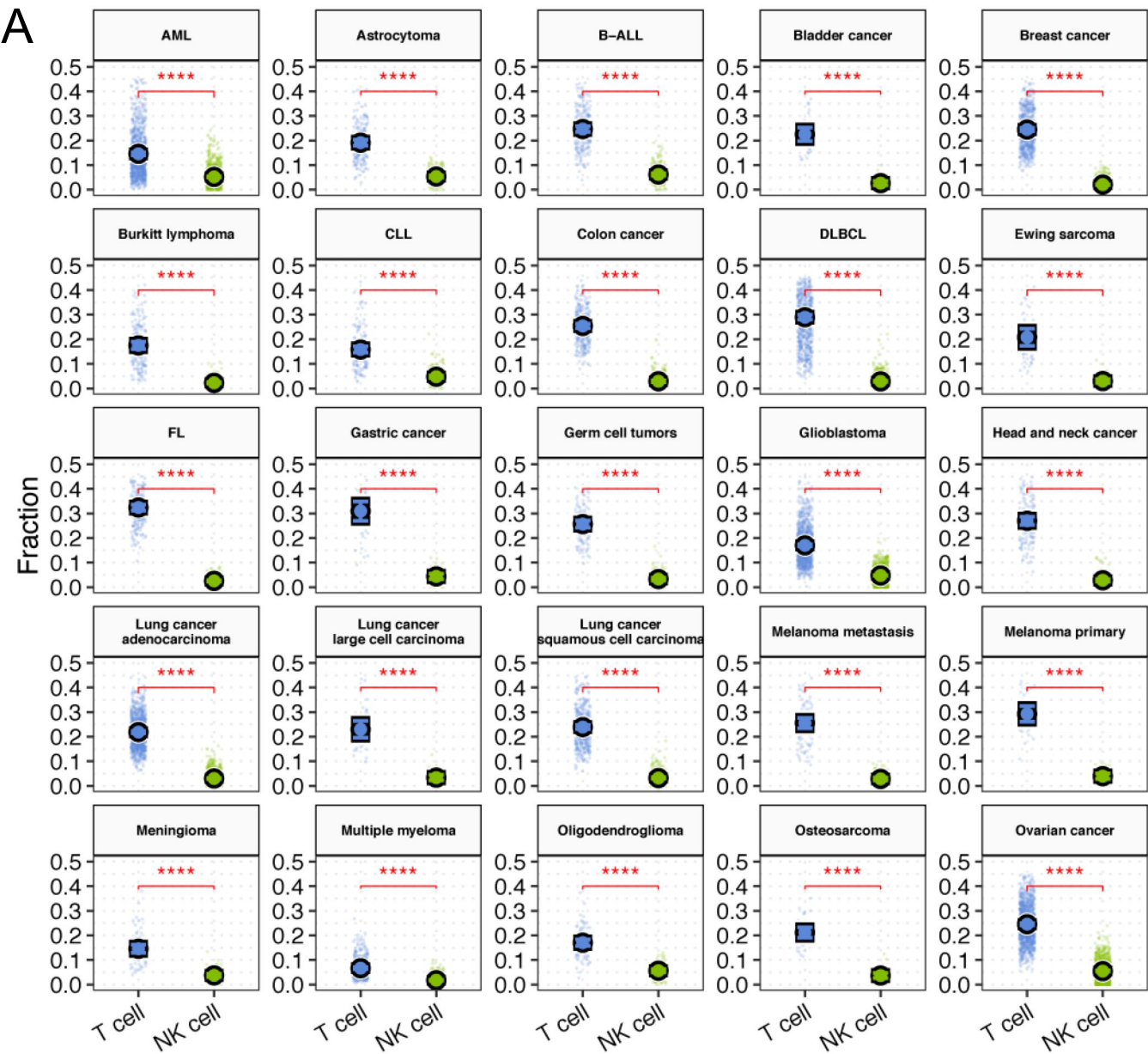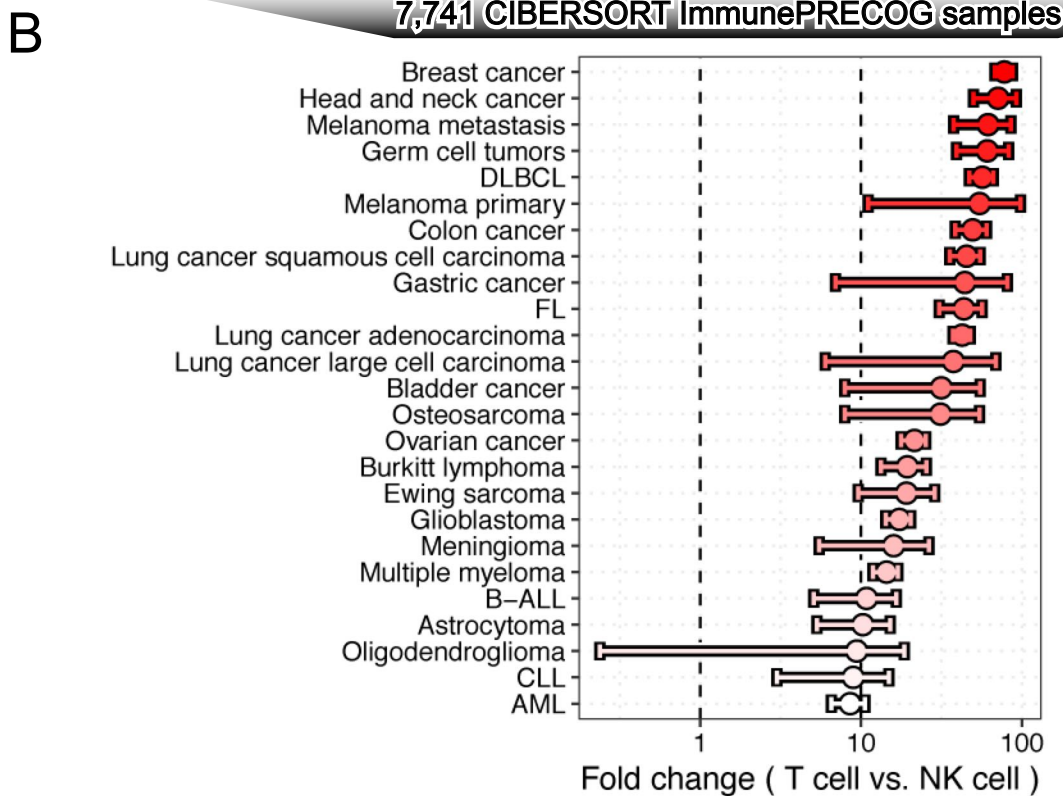

Supplement: Figure S1 — t-SNE projection of single-cell RNA-sequencing data from 28,823 human PBMCs, with each dot representing one single cell and colors representing the expression of known canonical marker genes (A) and 5 major cell lineages (B). [file DataSheet_1.zip › Supplementary_Figure_2.pdf]

# Supplementary Figure 1

A

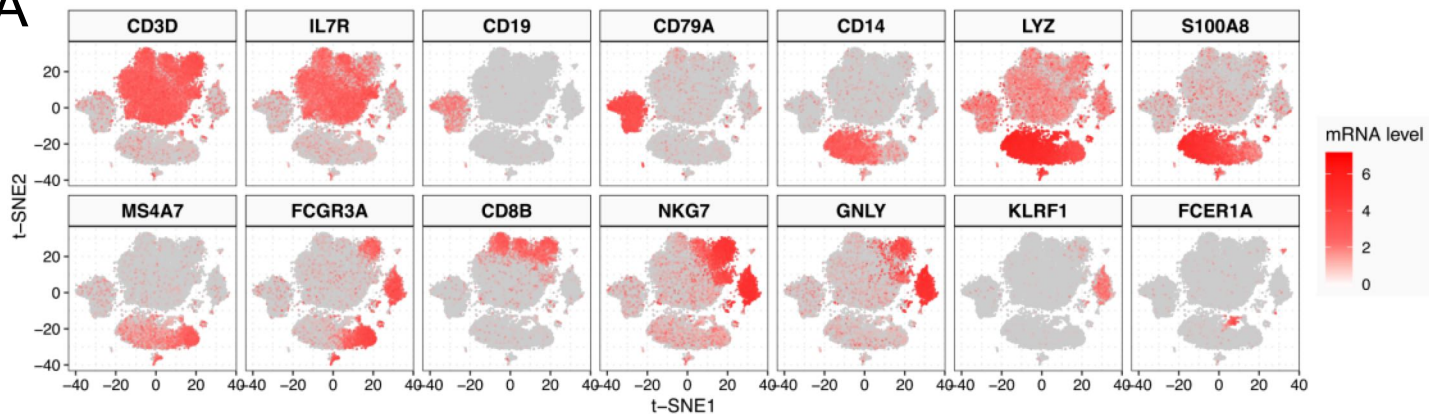

33,000 peripheral blood mononuclear cells (PBMC)

B

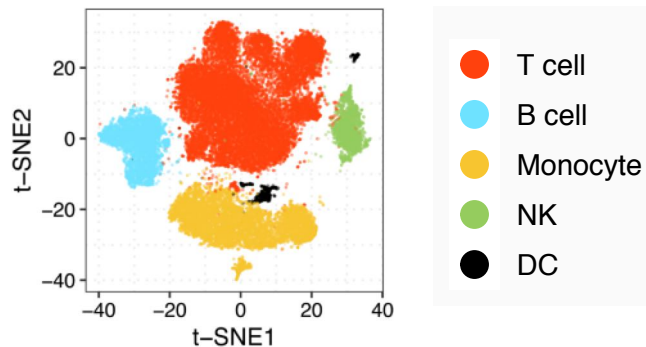

Supplement: Figure S1 — t-SNE projection of single-cell RNA-sequencing data from 28,823 human PBMCs, with each dot representing one single cell and colors representing the expression of known canonical marker genes (A) and 5 major cell lineages (B). [file DataSheet_1.zip › Supplementary_Figure_1.pdf]

# Supplementary Figure 5

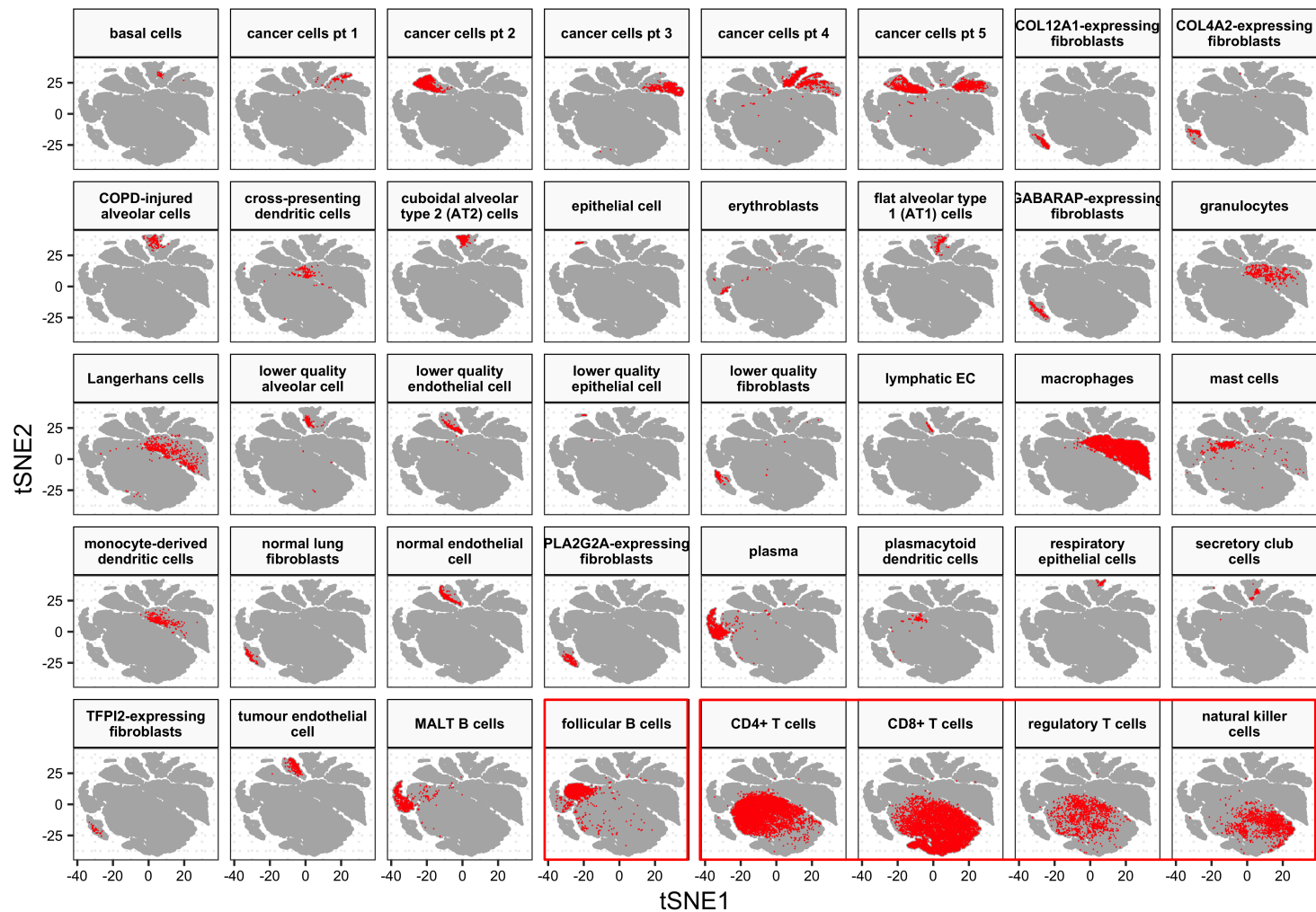

Supplement: Figure S1 — t-SNE projection of single-cell RNA-sequencing data from 28,823 human PBMCs, with each dot representing one single cell and colors representing the expression of known canonical marker genes (A) and 5 major cell lineages (B). [file DataSheet_1.zip › Supplementary_Figure_5.pdf]

# Supplementary Figure 4

A

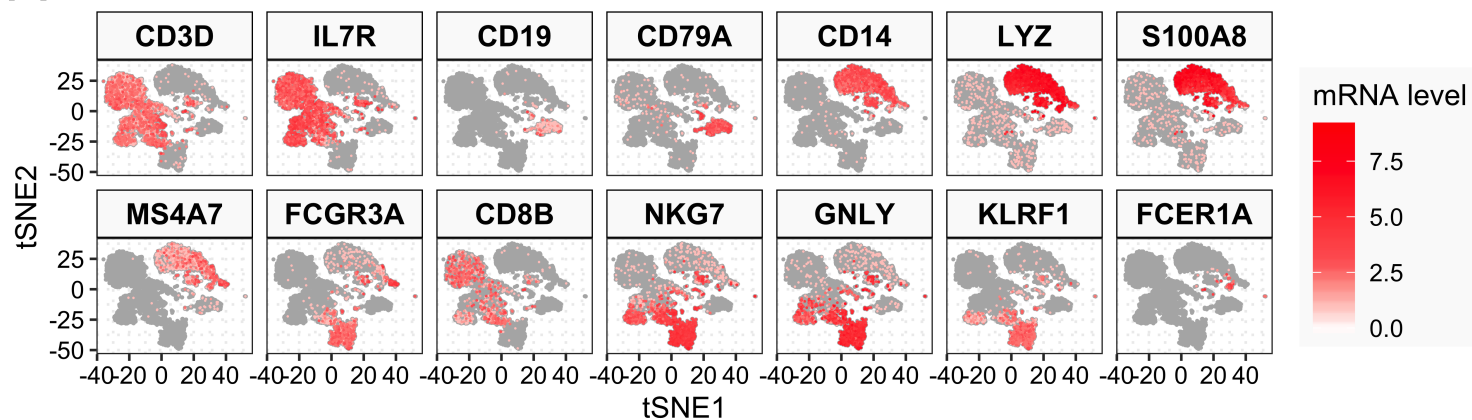

B

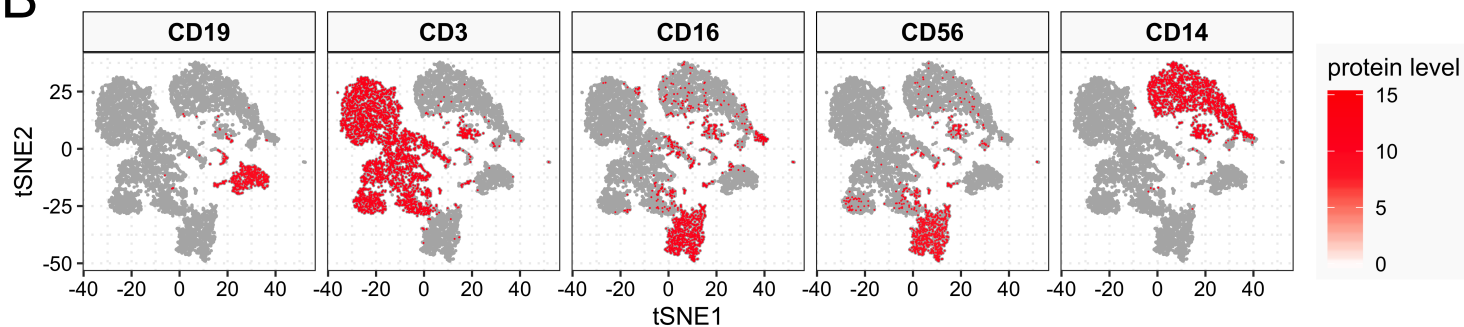

C

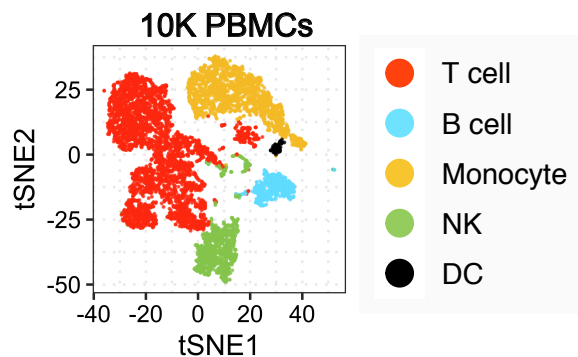

D

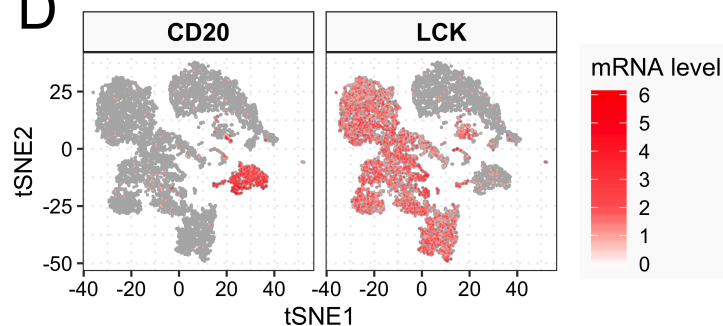

Supplement: Figure S1 — t-SNE projection of single-cell RNA-sequencing data from 28,823 human PBMCs, with each dot representing one single cell and colors representing the expression of known canonical marker genes (A) and 5 major cell lineages (B). [file DataSheet_1.zip › Supplementary_Figure_4.pdf]

# Supplementary Figure 6

A

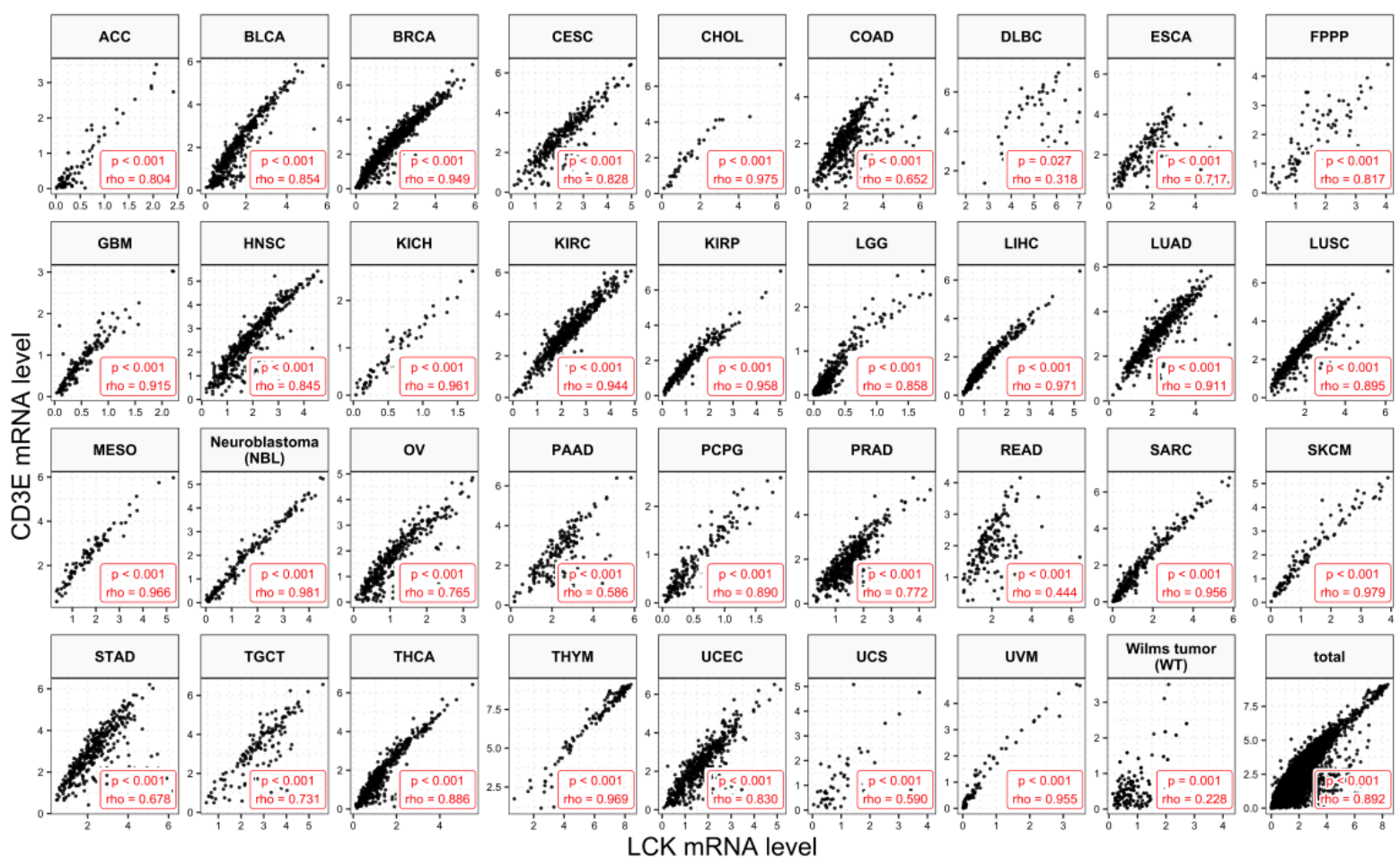

B

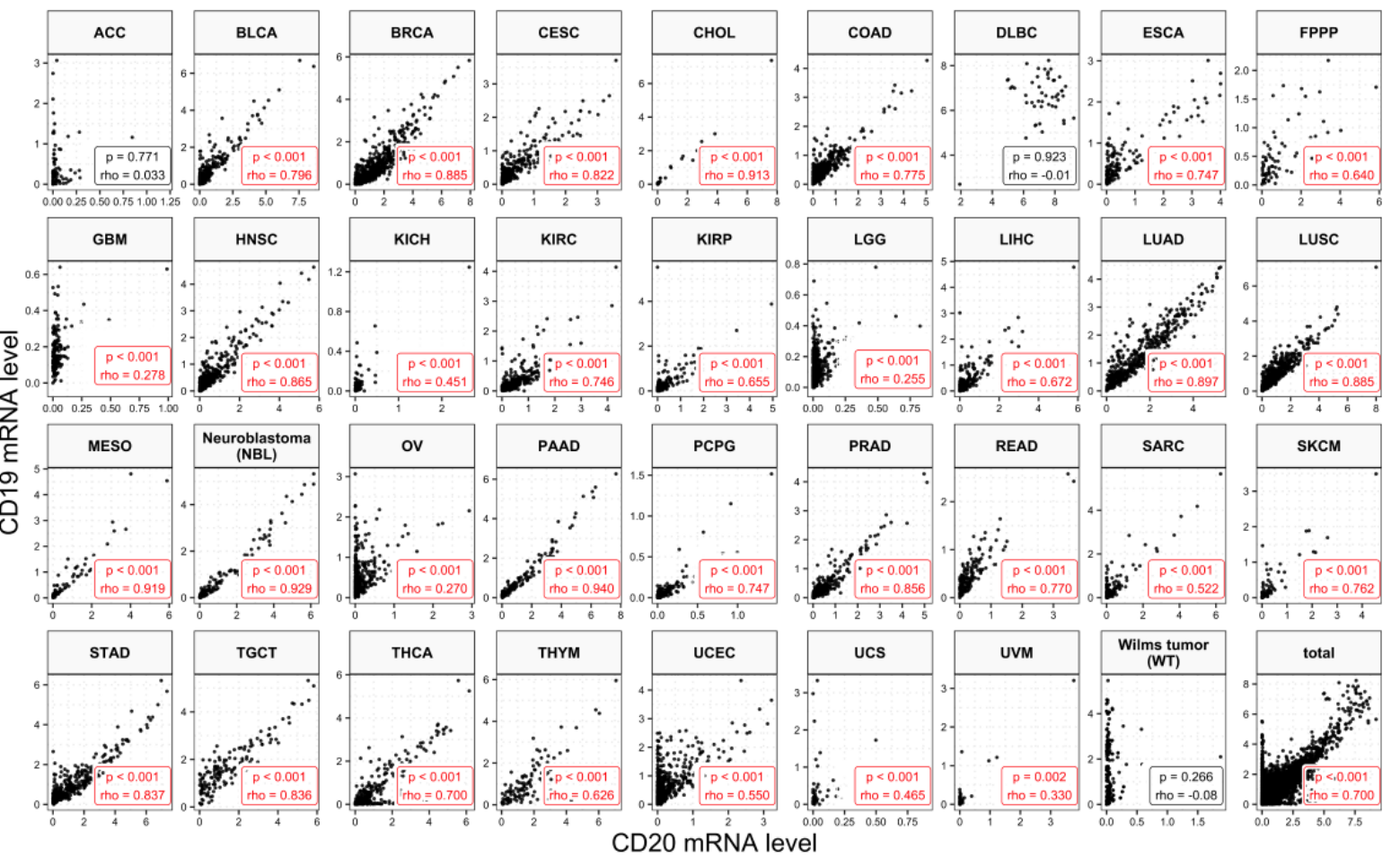

Supplement: Figure S1 — t-SNE projection of single-cell RNA-sequencing data from 28,823 human PBMCs, with each dot representing one single cell and colors representing the expression of known canonical marker genes (A) and 5 major cell lineages (B). [file DataSheet_1.zip › Supplementary_Figure_6.pdf]

# Supplementary Figure 7

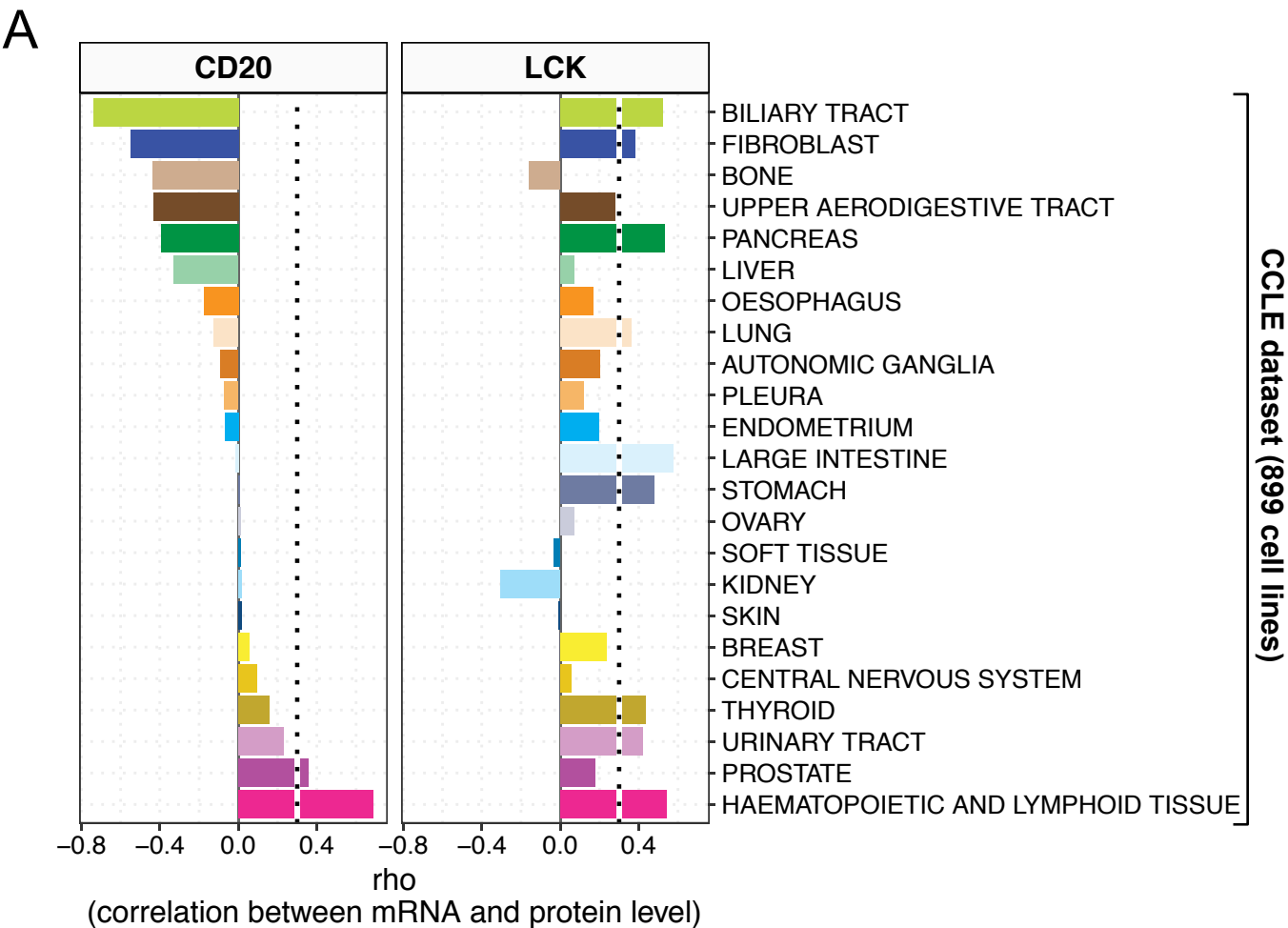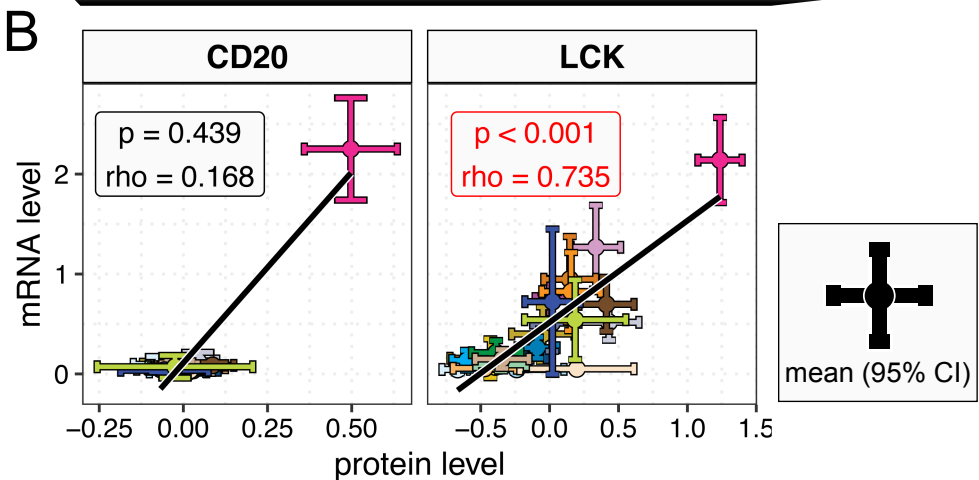

Supplement: Figure S1 — t-SNE projection of single-cell RNA-sequencing data from 28,823 human PBMCs, with each dot representing one single cell and colors representing the expression of known canonical marker genes (A) and 5 major cell lineages (B). [file DataSheet_1.zip › Supplementary_Figure_7.pdf]
